# Rapid Characterization of Point Defects in Solid-State Ion Conductors Using Raman Spectroscopy, Machine-Learning Force Fields, and Atomic Raman Tensors

**DOI:** 10.1021/jacs.4c07812

**Published:** 2024-09-18

**Authors:** Willis O’Leary, Manuel Grumet, Waldemar Kaiser, Tomáš Bučko, Jennifer L. M. Rupp, David A. Egger

**Affiliations:** † Department of Materials Science and Engineering, Massachusetts Institute of Technology, Cambridge, Massachusetts 02139-4307, United States; ‡ Department of Physics, TUM School of Natural Sciences, Technical University of Munich, Garching 85748, Germany; § Department of Physical and Theoretical Chemistry, Faculty of Natural Sciences, Comenius University Bratislava, Bratislava SK-84215, Slovakia; ∥ Institute of Inorganic Chemistry, Slovak Academy of Sciences, Bratislava SK-84236, Slovakia; ⊥ Department of Chemistry, TUM School of Natural Sciences, Technical University of Munich, Garching 85748, Germany; # Atomistic Modeling Center, Munich Data Science Institute, Technical University of Munich, Garching 85748, Germany

## Abstract

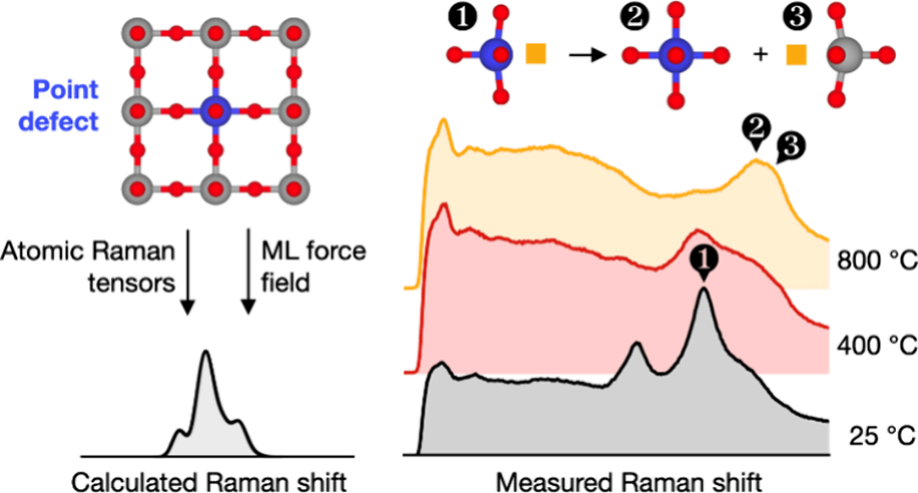

The successful design of solid-state photo- and electrochemical devices depends on the careful engineering of point defects in solid-state ion conductors. Characterization of point defects is critical to these efforts, but the best-developed techniques are difficult and time-consuming. Raman spectroscopy—with its exceptional speed, flexibility, and accessibility—is a promising alternative. Raman signatures arise from point defects due to local symmetry breaking and structural distortions. Unfortunately, the assignment of these signatures is often hampered by a shortage of reference compounds and corresponding reference spectra. This issue can be circumvented by calculation of defect-induced Raman signatures from first principles, but this is computationally demanding. Here, we introduce an efficient computational procedure for the prediction of point defect Raman signatures in solid-state ion conductors. Our method leverages machine-learning force fields and “atomic Raman tensors”, i.e., polarizability fluctuations due to motions of individual atoms. We find that our procedure reduces computational cost by up to 80% compared to existing first-principles frozen-phonon approaches. These efficiency gains enable synergistic computational–experimental investigations, in our case allowing us to precisely interpret the Raman spectra of Sr(Ti_0.94_Ni_0.06_)O_3-δ_, a model oxygen ion conductor. By predicting Raman signatures of specific point defects, we determine the nature of dominant defects and unravel impacts of temperature and quenching on *in situ* and *ex situ* Raman spectra. Specifically, our findings reveal the temperature-dependent distribution and association behavior of oxygen vacancies and nickel substitutional defects. Overall, our approach enables rapid Raman-based characterization of point defects to support defect engineering in novel solid-state ion conductors.

## 1 Introduction

Effectively engineering tomorrow’s energy materials requires a deep understanding of point defects, as these structural irregularities strongly impact functional properties and device performance. For example, point defects influence nonradiative recombination processes of charge carriers in solar materials,^1,2^ affect charge carrier mobilities in semiconductors,^3^ modulate thermal conductivity in metals,^4^ and accelerate material degradation.^5,6^ Ionic point defects play an especially critical role in solid-state ion conductors by providing migration pathways for mobile ions.^7,8^ Characterizing and engineering these defects—whose concentrations far exceed those found in other material classes^9^—is essential to enhancing solid-state ion conduction, which is itself among the key challenges in the design of high performance batteries,^10−15^ fuel cells,^16,17^ memristors,^18−21^ and opto-ionic devices.^22,23^ Manipulation of ionic point defects via doping^24−27^ has proven an especially powerful strategy, having led to orders-of-magnitude improvements in ionic conductivity across various material classes.^9,28−32^

Essential to engineering the properties of ionic point defects in solid-state ion conductors (and in materials in general) are methods to characterize the nature and concentration of these defects at finite temperatures. Common experimental methods to achieve this include positron annihilation lifetime spectroscopy,^33^ electron paramagnetic resonance spectroscopy,^34,35^ X-ray absorption/photoelectron spectroscopy,^27,36−38^ nuclear magnetic resonance spectroscopy,^39^ and vibrational spectroscopies—namely IR^40^ and Raman.^27,41,42^ Of these, Raman spectroscopy is particularly attractive due to its speed, flexibility, low cost, and widespread accessibility.^43^ It is particularly suited to characterization of solid-state ion conductors, as the high concentrations of ionic point defects give rise to detectable signatures within the Raman spectra of host materials. However, extracting information on ionic point defects from experimental Raman spectra poses enormous challenges.

To understand the precise origin of these challenges, we must first briefly review the mechanisms underlying Raman spectroscopy. Raman scattering of photons, on which Raman spectroscopy relies, arises from thermal motion of atoms and the effect these motions have on a material’s polarizability. Assuming approximately harmonic atomic motions and perfect crystallinity of the material, thermal atomic motion can be described using phonons, which correspond to normal-mode lattice vibrations. Polarizability, denoted here by **α**, is a second-order, symmetric tensorial material property relating polarization ***p*** and external electric field ***E***:
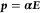
The atomic motions making up a given phonon perturb the distribution of electrons, generating minuscule fluctuations in the various components of **α.** The degree to which a phonon modulates **α** is known as its Raman tensor. Limiting our discussion to first-order Raman scattering, only Γ-point phonons with nonzero Raman tensors produce peaks in Raman spectra; these peaks appear at the frequencies of the Raman active phonons, and a peak’s intensity depends on the value of its corresponding Raman tensor.

For crystalline materials with perfect translational symmetry, the quantity and symmetries of the phonons and their corresponding Raman tensors can be readily determined purely from the crystallographic space group and atomic positions using group theory. By carrying out polarized Raman measurements at various orientations relative to a crystalline sample, these phonons, Raman tensors, and symmetries can be matched with the appropriate empirical Raman peaks.^44,45^ However, this theoretical framework is no longer valid in solid-state ion conductors containing substantial concentrations of point defects. Point defects break translational symmetry, meaning that the language of phonons, space groups, and symmetry operations no longer applies. Of course, the underlying mechanism of Raman scattering remains unchanged; Raman spectra of solid-state ion conductors still reflect thermal vibrations of atoms and their influence on the polarizability. Point defects, meanwhile, strongly affect thermal vibrations and the distribution of electrons. Hence, their presence leads to perturbations from a material’s defect-free Raman spectrum in the form of intensity changes, new peaks, peak shifts, and peak splitting.^44,46^ At sufficient defect concentrations, these perturbations can be experimentally resolved.

While many studies have taken advantage of these perturbations to characterize point defects in a wide range of bulk^20,47−51^ and 2D^52−54^ materials, the theoretical difficulties outlined above make it challenging to interpret and understand Raman spectra of defective materials. These difficulties are conventionally sidestepped by comparing Raman spectra of defective materials against those of prudently selected reference materials known to contain the point defects of interest. However, well-vetted reference spectra are rarely available for solid-state ion conductors, and developing libraries of reference materials/spectra can be tedious.

Alternatively, one can simulate a defective structure’s Raman signatures using density functional theory (DFT) calculations. This can be done through either static^55,56^ (frozen phonon) or dynamic^57,58^ (molecular dynamics) approaches. In both cases, two sets of calculations are performed per structure: one set to determine the atomic thermal motions and a proceeding set to characterize the influence these motions have on the system’s polarizability tensor. The results of these calculations are combined to yield a simulated Raman spectrum, from which signatures related to the point defects can be identified and compared to experimental measurements. Such calculations allow interpretation of empirical Raman spectra without the need to build a library of thoroughly characterized reference materials, all while providing a great degree of atomic-level physical insight.^59−63^ In theory, Raman spectroscopy offers excellent opportunities to synergize experiments and calculations to study complex, defective materials. In practice, however, the need to calculate numerous polarizability tensors, usually done with density functional perturbation theory^64,65^ (DFPT), makes conventional Raman calculations very computationally demanding. The computational cost can quickly become insurmountable, especially when treating complex and relevant solid-state ion conductors.

There is therefore sustained interest in reducing the computational cost of DFT-level Raman calculations. One such approach was derived by Lazzeri and Mauri, who introduced an efficient method of obtaining the Raman spectra using second order derivatives of the DFT density matrix. Use of this new formalism dramatically cuts down the required number of DFPT calculations.^66^ Other approaches reduce computational cost through estimation of polarizability tensors. For example, Hashemi et al. approximated Raman spectra of defective systems based on the vibrational and Raman properties of defect-free systems.^67^ Very recently, some of the present authors trained machine learning (ML) models to predict polarizability tensors from atomic positions; they showed that a relatively small training set, constructed from DFPT data, allowed prediction of Raman spectra with high accuracy.^68^ Still, calculating Raman spectra of defective systems with DFT poses many challenges. Since point defects break translational symmetry, large simulation cells are required to minimize spurious interactions between periodic images.^59^ Furthermore, the effects of defect-induced local structural distortions, as well as possible interactions between defects, must be carefully evaluated. Altogether, new computational approaches are urgently needed to augment Raman investigations of defective solid-state ion conductors. Advances in this area promise to enhance our mechanistic understanding of ionic point defects, deliver new and refined materials design criteria, and bring point defect screening to manufacturing and other high-throughput settings.

In this work, we present an efficient approach for calculating Raman spectra of ionic point defects in solid-state ion conductors. In our method, we use machine-learning force fields (MLFFs) to determine vibrational modes and calculate each mode’s influence on the polarizability tensor using “atomic Raman tensors”, which connect polarizability fluctuations to individual atomic displacements. This allows us to predict and identify the spectral signatures of point defects in experimental Raman spectra. We demonstrate the synergistic capabilities and advantages of our methodology by analyzing the Raman spectra of a model material: Ni-doped SrTiO_3_ (STO), i.e., Sr(Ti_1–*x*_Ni_*x*_)O_3-δ_ (STN). STN is a mixed electronic-ionic conductor,^69^ promising photocatalyst,^70^ and (with the substitution of La onto the Sr site) an excellent solid oxide fuel cell anode.^71^ STN, like other members of the perovskite family,^72^ exhibits a rich defect chemistry; introduction of Ni^2+^ ions onto the Ti^4+^ site leads to Ni_Ti_^″^ substitutional point defects, whose charge is compensated by oxygen vacancies, V_O_^••^, which facilitate oxygen ion conduction. Furthermore, it is well-established that acceptor dopants in titanates, like Ni^2+^, can trap V_O_^••^, resulting in immobile defect associates (e.g., (Ni_Ti_^″^–V_O_^••^)^×^) that reduce the overall O^2–^ conductivity at low temperatures (Figure S1).^73−80^ However, previous literature reports have been unable to convincingly resolve the Raman signatures of these point defects in SrTiO_3_ (Supporting Information Section S.1). Our joint computational and experimental results, on the other hand, reveal distinct Raman signatures arising from the presence of Ni_Ti_^″^ and V_O_^••^. Furthermore, we confirm the temperature-dependent association behavior of Ni_Ti_^″^ and V_O_^••^ and inhomogeneous Ni distribution within our samples. Through this, we demonstrate how our approach enables Raman characterization of point defects with modest computational cost. We conclude with a discussion of the potential and current limitations of our approach, particularly when applied to a wider class of materials. Overall, we believe that our method makes Raman spectroscopy a more powerful and attractive tool to characterize and tailor point defects in solid-state ion conductors for batteries, fuel cells, and beyond.

## 2 Computational Methodology

### 2.1 Construction of Atomistic Models and Calculation of Vibrational Modes

**Figure 1:**
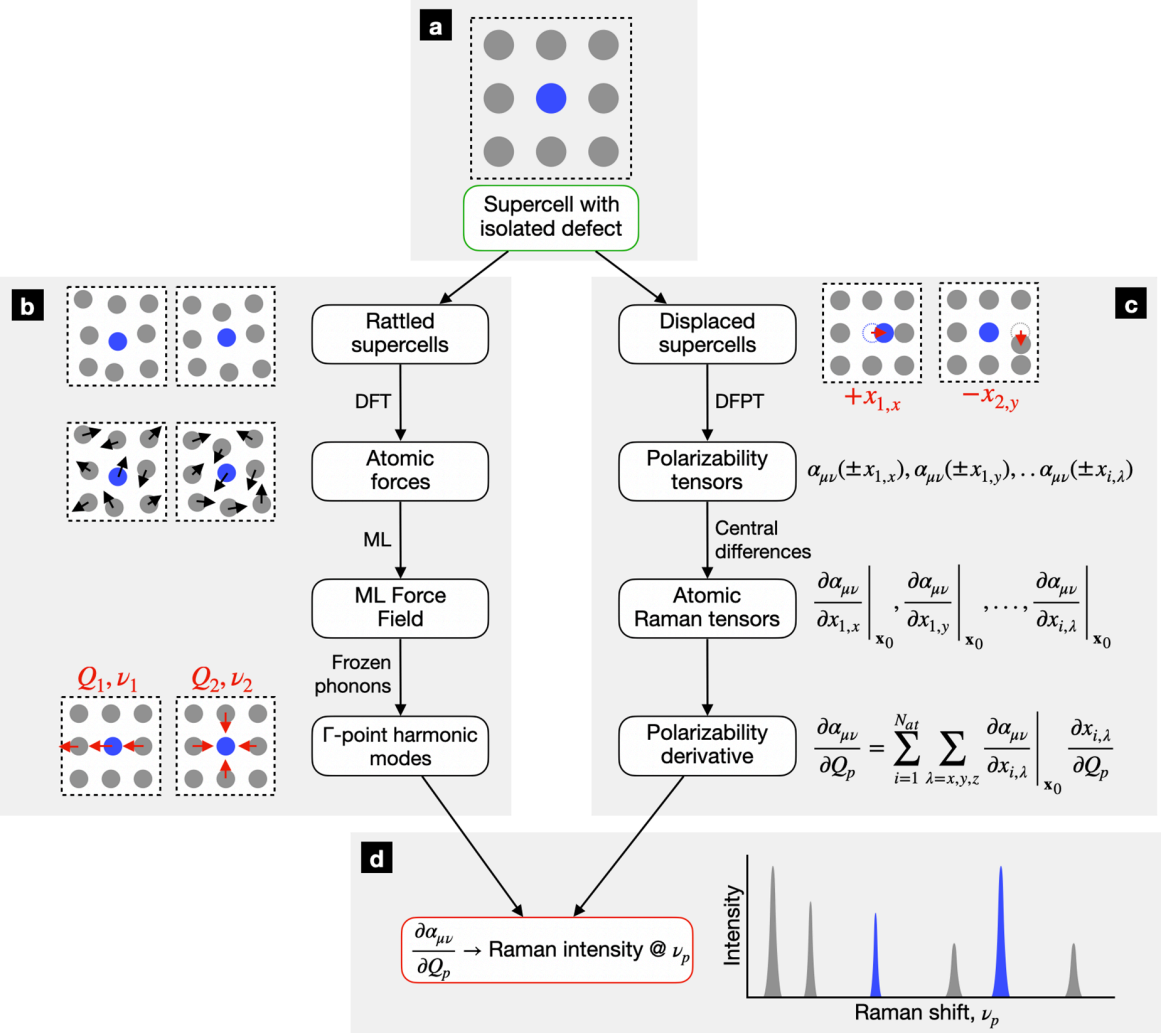
High-level description of our methodology to compute Raman signatures of ionic point defects in crystalline materials. (a) A supercell containing the point defect of interest is constructed. (b) The supercell’s Γ-point harmonic vibrational modes are calculated using a machine-learning (ML) force field trained on “rattled” supercells. (c) The atomic Raman tensors are evaluated with central differences and density functional perturbation theory (DFPT). Polarizability directional derivatives are derived using atomic Raman tensors. (d) Raman intensities are calculated using the polarizability directional derivatives.

The identities and precise arrangements of ionic point defects in a material are often unknown, making it challenging to construct computationally feasible atomistic models that mirror experimental reality. To identify the contribution of individual ionic point defects in finite-temperature Raman spectra, we separately treat candidate ionic point defects using a supercell approach (Figure 1a).^59^ Although this approach neglects interactions between point defects, regions nearest to a given defect—vibrations within which are responsible for the majority of the defect’s Raman signature—are well approximated. As we will later show, this supercell approach is highly advantageous when computing Raman spectra.

We next calculate the Γ-point vibrational modes of the supercell using the frozen phonon method. These calculations become computationally demanding when modeling point defects, whose presence requires the use of larger, lower-symmetry simulation cells compared to those needed to model defect-free materials. We reduce the computational cost of frozen phonon calculations by leveraging MLFFs, a powerful and general method that has recently gained attention for calculating accurate vibrational properties (Figure 1b).^81−84^ As with all frozen phonon calculations, we begin by fully relaxing a defect-containing supercell with DFT. We then produce several stochastically perturbed versions of this relaxed structure using a Monte Carlo-based “rattling” procedure.^82^ Atomic forces for each of these perturbed structures are calculated with DFT. An MLFF is trained on these forces; in our case, we employ a kernel-based ML model^85−87^ implemented in VASP v.6.3.0.^88−91^ Finally, we carry out a frozen phonon calculation on the relaxed structure using the MLFF, which provides the frequencies and eigendisplacements of the vibrational modes at the Γ-point at negligible computational cost. The use of frozen-phonon calculations is only valid in systems with meaningful normal modes, i.e., where harmonic oscillations around the potential energy minimum dominate the system dynamics. These limitations could be lifted, for example by accounting for instantaneous normal modes^92,93^ around nonminimum positions, but this is beyond the scope of this study.

### 2.2 Calculation of Per-Mode Raman Intensities

To construct first-order Raman spectra, we calculate Raman intensities for the vibrational modes.^44,94^ The key physical quantity determining the Raman intensity of a mode *p* with normal coordinate *Q*_ *p*_ is the directional derivative *∂α*_*μν*_/*∂Q*_ *p*_.^95^*α*_*μν*_ (μ = *x*, *y*, *z*; ν = *x*, *y*, *z*) is a component of the polarizability tensor **α**, which for our purposes is interchangeable with the dielectric tensor *ϵ*_*μν*_. Conventionally, *∂α*_*μν*_/*∂Q*_ *p*_ is directly calculated with central differences, requiring two polarizability calculations for each of the 3(*N*_*at*_ – 1) modes, where *N*_*at*_ is the number of atoms in the supercell. This implies that a total of 6(*N*_*at*_ – 1) polarizability calculations are needed. In practice, a small handful of these modes may be degenerate due to symmetry, possessing symmetrically equivalent *Q*_ *p*_’s and therefore *∂α*_*μν*_/*∂Q*_ *p*_’s. Taking advantage of these symmetries, one can moderately reduce the number of polarizability calculations. Still, since space groups only give rise to one-, two-, or three-dimensional irreducible representations, only one-, two-, and three-fold degeneracies are allowed. Therefore, when carrying out conventional Raman calculations with structures displaced along normal coordinates, a minimum of 2(*N*_*at*_ – 1) polarizability calculations are required to derive a complete Raman spectrum.^96^

Rather than directly computing ∂α_μν_/∂*Q*_ *p*_ by displacing atoms along the normal coordinates, we instead determine the directional derivative using the gradient of α_μν_ evaluated in the atomic Cartesian coordinate basis. Defining *x*_*i*,λ_ as atom *i*’s Cartesian coordinate in the λ = *x*, *y*, *z* direction, ∂α_μν_/*∂Q*_ *p*_ can be expressed as
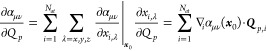
1where ***x***_0_ denotes the atomic coordinates of the equilibrium structure, **∇**_*i*_ is the gradient operator defined with respect to the Cartesian coordinates of atom *i*, and ***Q***_*p*,*i*_ ≡ ∂***x***_*i*_/∂*Q*_ *p*_. Using eq 1 to compute Raman intensities requires calculating the coefficients *∂α*_μν_/*∂x*_*i*,λ_, which we dub the “atomic Raman tensors”. Within the harmonic approximation, the observable (phonon) Raman tensors are simply linear combinations of these atomic Raman tensors. To calculate each *∂α*_μν_/∂*x*_*i*,λ_, we construct two “displaced” supercells with atom *i* displaced slightly in the ±λ directions. The polarizabilities for both displaced supercells are computed, and the atomic Raman tensor is subsequently calculated using central differences (Figure 1c). Note that the application of the chain rule in eq 1 is conceptually similar to approaches used in the prediction of infrared spectra from atomic polar tensors and effective charges,^97,98^ with the difference being that atomic Raman tensors concern polarizability instead of polarization.

By applying eq 1, we are essentially evaluating ∂α_μν_/*∂Q*_ *p*_ in the atomic coordinate basis, as opposed to the conventional evaluation of ∂α_μν_/∂*Q*_ *p*_ in the normal mode basis. The possibility of evaluation in the atomic coordinate basis has long been recognized,^99,100^ but evaluation within the normal mode basis remains the conventional strategy.^55,101^ Naively, one could expect that using eq 1 requires polarizability calculations for a total of 6*N*_*at*_ displaced supercells, i.e., six *more* than the conventional approach. However, translational invariance of **α** in periodic systems implies that , reducing the required polarizability calculations to 6(*N*_*at*_ – 1). Furthermore, any symmetry present in the relaxed supercell will imply crystallographic equivalence between some of the displaced supercells. Therefore, we can trivially relate their polarizability tensors using symmetry operations. In this way, the required number of polarizability calculations can be further reduced. In high symmetry systems–which have many symmetrically equivalent atoms and, by extension, atomic Raman tensors–this number can fall *below* the 2(*N*_*at*_ – 1) limit inherent to the conventional normal-mode-based approach.

Alongside more efficiently leveraging structural symmetries, use of eq 1 offers additional flexibility to reduce computational cost compared to the normal-mode-based approach, particularly when studying point defects in solid-state ion conductors and other materials. In a system containing point defects, many atoms may have local environments closely resembling those found in the defect-free structure. Atomic Raman tensors from the defect-free structure can therefore be “inherited” for these atoms without significant loss in accuracy. Furthermore, since eq 1 explicitly captures the connections between the motions of individual atoms and the computed Raman intensity, one can limit investigation to the atoms most relevant to the research question at hand. If a specific frequency range is of interest, for example, only atomic Raman tensors for atoms that move within that frequency range need to be considered. Furthermore, if the vibrations of a particular local structure are of interest, only atomic Raman tensors for this local structure need to be considered, yielding a partial Raman spectrum. Although one must be careful when interpreting experimental data with such partial spectra, we will now demonstrate that this approach allows straightforward isolation of the Raman signatures arising from point defects in solid-state ion conductors.

## 3 Results

### 3.1 Calculation of Raman Signatures of Ionic Point Defects in Ni-Doped SrTiO_3_

**Figure 2:**
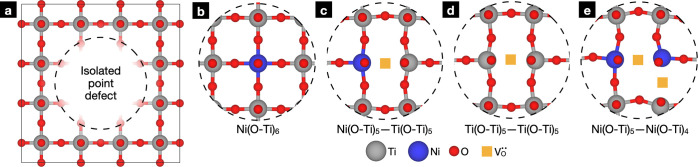
(a) Schematic of the supercell used to model point defects in SrTiO_3_. (b), (c), (d), and (e) show the relaxed geometries of Ni_Ti_^″^, (Ni_Ti_^″^–V_O_^••^)^×^, V_O_^••^, and (Ni_Ti_^″^–V_O_^••^–Ni_Ti_^″^–V_O_^••^)^×^, respectively. Sr atoms are not pictured for clarity.

Ni_Ti_^″^, V_O_^••^, and defect associates thereof are highly relevant to oxygen ion conduction in STN. To predict how the interaction between Ni_Ti_^″^ and V_O_^••^ would manifest itself in the Raman spectra of STN, we applied our methodology to four distinct point defects contained in 4 × 4 × 4 STO supercells (Figure 2). Although STO is expected to adopt a tetragonal structure at 0 K,^102^ we enforce a cubic supercell based on the long-range cubic symmetry of our experimental STN samples (to be presented and discussed later in Section 3.2). We first considered Ni_Ti_^″^ and V_O_^••^ separately, then investigated the point defect associate (Ni_Ti_^″^–V_O_^••^)^×^. Finally, we considered a complex of two such defect associates, (Ni_Ti_^″^–V_O_^••^–Ni_Ti_^″^–V_O_^••^)^×^. Here, one V_O_^••^ is stabilized between two Ni_Ti_^″^ defects,^103^ while the second V_O_^••^ is only coordinated to one Ni_Ti_^″^. We considered this point defect complex based on the possibility of inhomogeneous distribution of acceptor dopants in STO, particularly near grain boundaries,^104−107^ which could give rise to Ni-enriched regions. Setting aside the aim to accurately capture the precise structure of Ni-enriched regions, this defect complex may still give relevant insights into the vibrational signatures that arise from structures within such regions.

Inclusion of a single Ni_Ti_^″^ results in a Ni(O–Ti)_6_ unit which did not appreciably perturb the geometry of nearby atoms (Figure 2b), suggesting that the Ni^2+^ ion behaves similarly to Ti^4+^. Still, we expected vibrations of the oxygen atoms within the Ni–O–Ti motifs to be highly Raman active due to local asymmetry in the electron density. For (Ni_Ti_^″^–V_O_^••^)^×^ and V_O_^••^, we observed a substantial distortion of nearby atoms, forming adjacent Ni(O–Ti)_5_ and/or Ti(O–Ti)_5_ units that buckled toward the coordinating V_O_^••^ (Figure 2c,d). Due to these distortions, we expected oxygen atoms bonded to undercoordinated B-site cations to contribute to Raman activity; again, we expected oxygens contained in the Ni(O–Ti)_5_ unit to be particularly Raman active due to asymmetries in the electron density within the Ni–O–Ti motifs. The presence of (Ni_Ti_^″^–V_O_^••^–Ni_Ti_^″^–V_O_^••^)^×^ distorted the oxygen sublattice further (Figure 2e), forming Ni(O–Ti)_5_, Ni(O–Ti)_4_, and Ti(O–Ti)_5_ units. We similarly expected these units to contribute substantially to the Raman spectrum. In this case, these units introduced long-range distortions in the supercell in which B-site octahedra tilted in the plane occupied by the two Ni atoms.

For the four supercells, we trained MLFFs using 20 distorted training structures each. We then applied the frozen phonon method using these MLFFs and evaluated Raman intensities using eq 1. Details on these steps can be found in Supporting Information Section S.3. In addition, we verified the accuracy of the vibrational frequencies obtained from the MLFF against DFT calculations (Supporting Information Section S.4). This demonstrates that kernel-based MLFFs can yield vibrational properties at low cost.

We then calculated Raman spectra for each of our four supercells employing atomic Raman tensors as implemented in the ramannoodle package.^108^ When determining the atomic Raman tensors ∂α_μν_/∂*x*_*i*,λ_, three key considerations greatly reduced the required number of DFPT polarizability tensor calculations. First, we observed that vibrations above 600 cm^–1^ were dominated by motion of light oxygen atoms (Figure S5). We therefore decided to focus solely on the Raman spectra above 600 cm^–1^. Under our approach, this choice allowed us to focus exclusively on ∂α_μν_/∂*x*_*i*,λ_’s associated with oxygen atoms, which we calculated using 0.16 and 0.26 Å displacements parallel and orthogonal to the Ti–O/Ni−O bonds, respectively. Second, we fully exploited the symmetries in the relaxed supercells, using them to reduce the number of explicit polarizability tensor calculations. Third, and most critically, we noted that the presence of Ni_Ti_^″^, V_O_^••^, and (Ni_Ti_^″^–V_O_^••^)^×^ did not substantially distort the STO lattice far away from the defects. Therefore, we hypothesized that the ∂α_μν_/∂*x*_*i*,λ_’s of weakly perturbed atoms far away from these point defects could be inherited from those of perfect STO, in which all ∂α_μν_/∂*x*_*i*,λ_’s are zero by symmetry. Explicit comparison showed that the sole inclusion of ∂α_μν_/∂*x*_*i*,λ_’s of the oxygen atoms within the Ni(O–Ti)_*x*_ and Ti(O–Ti)_5_ units was sufficient for obtaining converged Raman spectra, confirming our hypothesis (Supporting Information Section S.6). Here, we note that the supercell containing (Ni_Ti_^″^–V_O_^••^–Ni_Ti_^″^–V_O_^••^)^×^ showed a non-negligible distortion even for oxygen atoms far away from the defect. In this case, we view this distortion as an artifact of the finite size of the supercell. We therefore chose to only include the oxygen ∂α_μν_/∂*x*_*i*,λ_’s of the Ni(O–Ti)_*x*_ and Ti(O–Ti)_5_ units, in effect isolating the contributions of the point defects.

**Figure 3:**
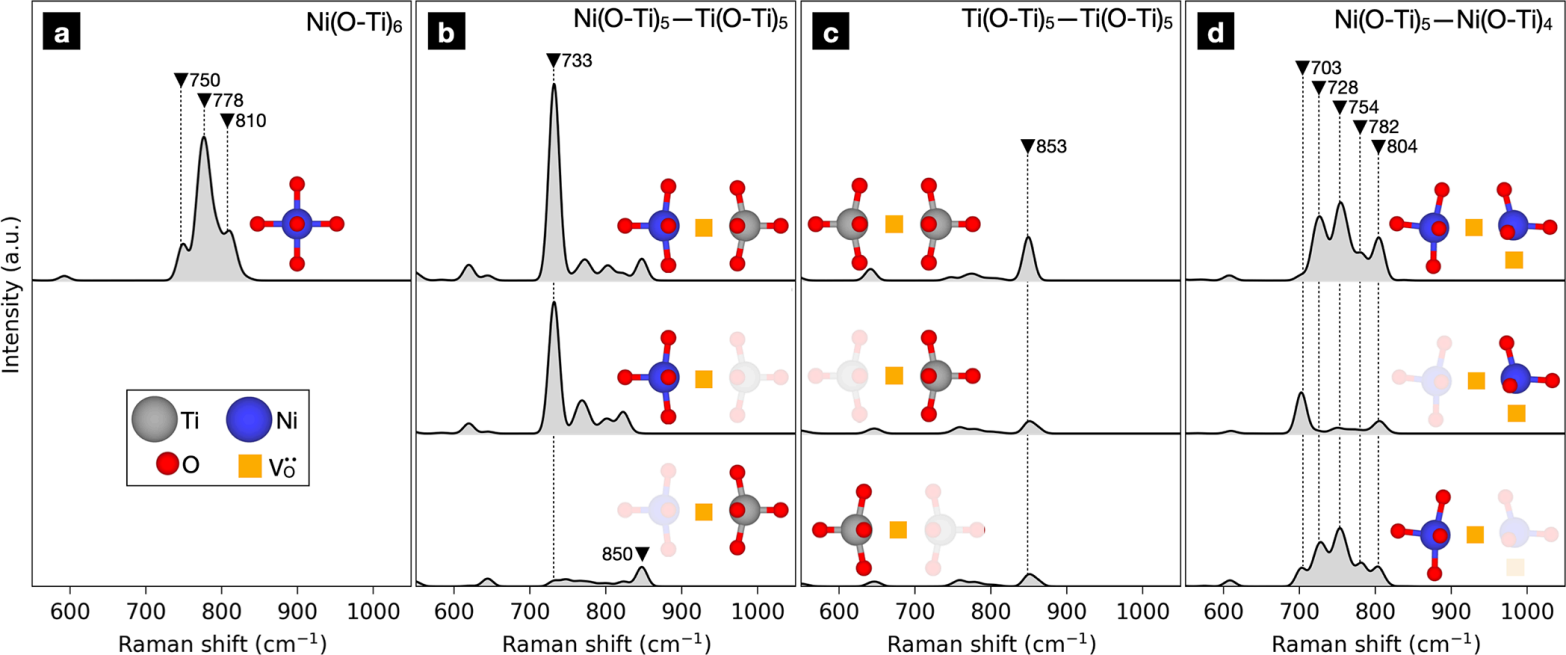
Calculated Raman signatures of (a) Ni_Ti_^″^, (b) (Ni_Ti_^″^–V_O_^••^)^×^, (c) V_O_^••^, and (d) (Ni_Ti_^″^–V_O_^••^–Ni_Ti_^″^–V_O_^••^)^×^. The Raman shifts of major Raman bands are labeled in units of cm^–1^. Total spectra are presented above, while partial spectra associated with specific structural units are shown below. To aid in visualization, discrete peaks were convoluted by Gaussians with standard deviation of 7 cm^–1^. The spectra have a common normalization to allow a quantitative comparison of Raman signatures between different point defects.

The local distortions and asymmetry that arise from the introduced point defects gave rise to strong, first-order Raman signatures for all considered defects (Figure 3). It is essential to highlight that no first-order Raman-active vibrations exist in pristine STO, implying that all first-order Raman features in the defective systems are entirely due to the presence of the point defects. We examined the corresponding eigendisplacements for each major peak and found that they all involve breathing vibrations of the various local structural units, i.e., Ni(O–Ti)_*x*_ and Ti(O–Ti)_5_.

The frequencies of the structure units’ harmonic breathing modes depended strongly on their composition. The Ni(O–Ti)_6_ unit gave rise to a strong band at 778 cm^–1^ alongside weaker shoulder bands at 750 and 810 cm^–1^ (Figure 3a). (Ni_Ti_^″^–V_O_^••^)^×^ induced a strong Raman band at 733 cm^–1^ due to the Ni(O–Ti)_5_ unit (Figure 3b). The Ti(O–Ti)_5_ unit within this defect gave rise to a Raman band at 850 cm^–1^. However, this band was relatively weak compared to Ni(O–Ti)_5_’s signature. This is because of the significant asymmetry in the charge density between the Ni–O and Ti–O bonds of the Ni–O–Ti unit. Meanwhile, an isolated V_O_^••^ generated a weak Raman band at 853 cm^–1^, which arose from oxygen vibrations of the two Ti(O–Ti)_5_ units (Figure 3c). The signature of this band is close to the Ti(O–Ti)_5_’s signature within (Ni_Ti_^″^–V_O_^••^)^×^, demonstrating that a nearby Ni(O–Ti)_5_ unit does not substantially change the Raman signature of a Ti(O–Ti)_5_ unit.

For (Ni_Ti_^″^–V_O_^••^–Ni_Ti_^″^–V_O_^••^)^×^, we observed a broad, multipeak feature between 703 and 804 cm^–1^ (Figure 3d). Most of this band was derived from the Ni(O–Ti)_5_ unit which produced a broad feature peaking at 754 cm^–1^. This feature was far broader than that generated by the Ni(O–Ti)_5_ unit in (Ni_Ti_^″^–V_O_^••^)^×^ (Figure 3b), likely due to the substantial distortion induced by a very high concentration of point defects. Meanwhile, the Ni(O–Ti)_4_ subunit led to a Raman active band at 703 cm^–1^. This mode had a high Raman intensity in isolation but was obfuscated in the total spectrum due to coupling of the vibration with the adjacent Ni(O–Ti)_5_ unit. This indicates that the appearance of a major Ni(O–Ti)_4_ Raman signature is highly dependent on its arrangement with respect to other Ni-containing point defects. Note that in these calculations, we did not account for the atomic Raman tensors of the adjacent Ti(O–Ti)_5_. This is because movement of oxygens within this unit have a very weak Raman signature in comparison with those within the Ni-containing structural units (Figure 3b).

We finally emphasize that the overall computational cost of our methodology was roughly 80% less than traditional frozen-phonon-based Raman calculations (see Supporting Information Section S.8). These improvements are thanks in part to our use of MLFFs, which reduced the number of force calculations needed to obtain phonon properties. However, most computational savings were due to our evaluation of polarizability derivatives within the atomic coordinate basis, which greatly reduced the number of DFPT calculations required to obtain accurate Raman signatures. Here, we note that in some material systems the introduction of a single point defect in a supercell may break all preexisting symmetries, reducing our approach’s efficiency gains. Furthermore, our focus on high frequency vibrations allowed us to only consider oxygen atoms; calculation of STN spectra down to lower frequencies would require additional terms for Sr, Ti, and Ni. Even so, our methodology could be readily extended to overcome these limitations. Two atoms in similar local environments, even those that are not crystallographically equivalent, should give rise to nearly identical atomic Raman tensors. By identifying atoms in nearly identical local environments (using a quantitative metric and a cutoff, for example), the computational advantages of our approach could be realized in an even wider range of relevant material systems.

### 3.2 Temperature-Induced Changes in Ni-Doped SrTiO_3_’s Raman Spectrum

We used our computational predictions to interpret *in situ* and *ex situ* Raman spectra measured on a polycrystalline Sr(Ti_0.94_Ni_0.06_)O_3-δ_ (STN06) pellet. Critical experimental details will be presented here, while more detailed information can be found in Supporting Information Section S.9 and a previous publication.^109^ To prepare the pellet, STN06 powder was synthesized via a solid-state route by calcination of ball-milled powders at 1400 °C for 10 h under air with a 10 °C/min heating rate. X-ray diffraction confirmed formation of the cubic perovskite structure with traces of NiO impurities. The measured lattice constant (3.902 Å) was very close to that of pure STO (3.905 Å). Based on DFT-calculated lattice constants, this lack of change suggests roughly equal populations of Ni_Ti_^″^ substitutional defects and oxygen vacancies (Supporting Information Section S.10). From this powder, a pellet was pressed and sintered at 1500 °C for 10 h with a 10 °C/min heating rate.

**Figure 4:**
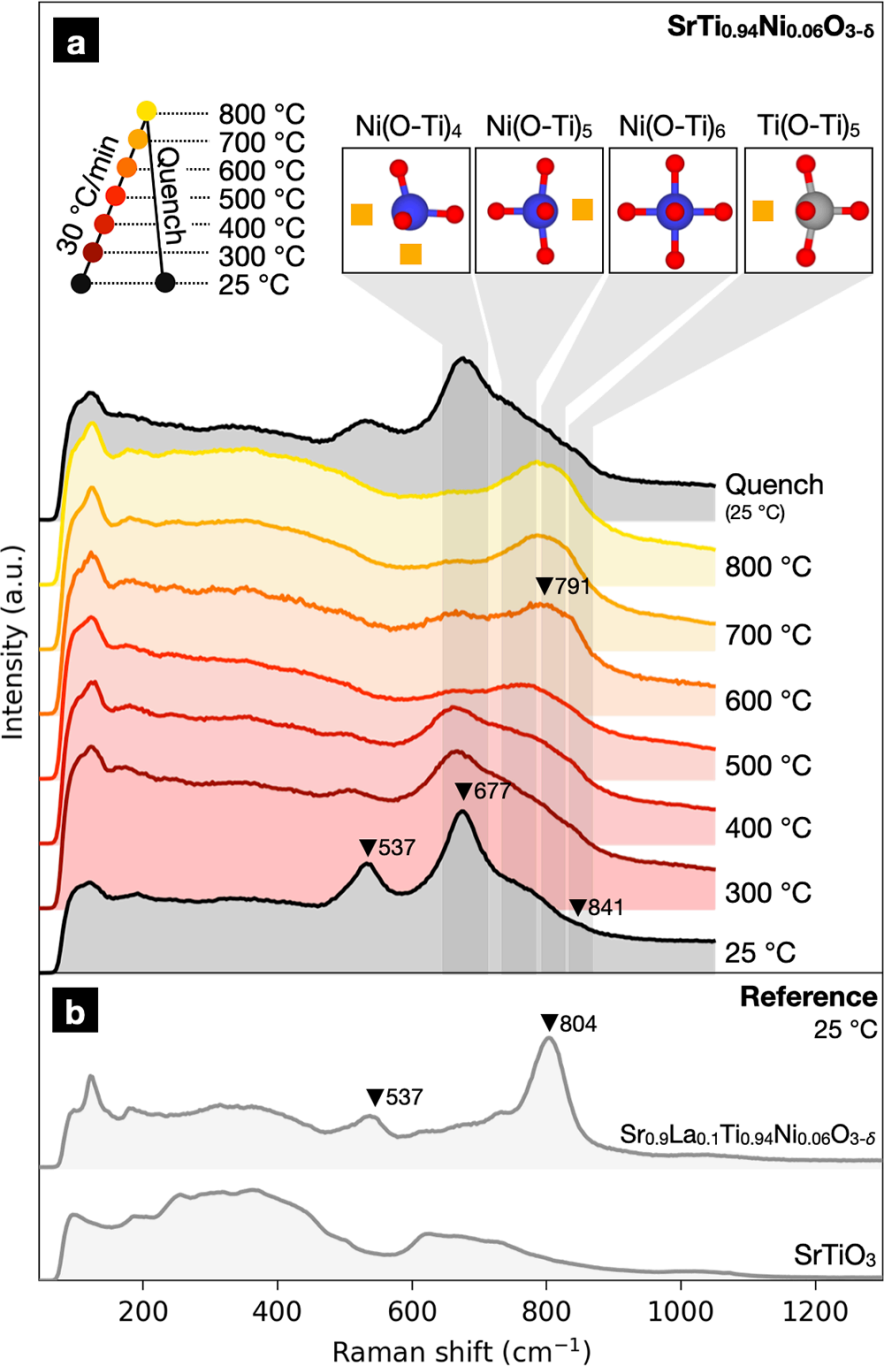
(a) Experimental *in situ* Raman spectra of Sr(Ti_0.94_Ni_0.06_)O_3-δ_ measured between 25 and 800 °C with a heating rate of 30 °C/min. Substantial changes in the spectra occurred above 500 cm^–1^, with the changes above this Raman shift indicative of the disassociation of oxygen vacancies trapped around Ni substitutional defects. (b) Reference spectra of SrTiO_3_ powder and a Sr_0.9_La_0.1_Ti_0.94_Ni_0.06_O_3-δ_ pellet measured at room temperature.

For this study, the STN06 pellet was broken apart manually, yielding several shards for use in our experiments. We first performed *in situ* Raman measurements of a STN06 shard under air at temperatures ranging from 25 to 800 °C. For this experiment, we selected a shard sourced from within the pellet interior. The spectra gathered over the course of this experiment are shown in Figure 4a alongside relevant reference spectra in Figure 4b. Pristine STO has no first-order Raman activity, meaning that STO’s Raman spectrum is entirely due to higher-order Raman scattering. STN06’s Raman spectrum below 500 cm^–1^, meanwhile, was considerably flatter than that of pure STO, which we attribute to point-defect-induced disorder. Although changes below 500 cm^–1^ were difficult to quantify, STN06 exhibited several well-defined Raman bands above 500 cm^–1^ that were absent in pure STO. Substitution of Ni led to strong bands at 537 and 677 cm^–1^ accompanied by a broad shoulder band from 739 to 770 cm^–1^ and a very weak band at 841 cm^–1^. Based on our calculations, we assign the strong band at 677 cm^–1^ to the presence of Ni(O–Ti)_4_ structural units (calculated Raman shift: 703 cm^–1^). We assign the broad shoulder from 739 to 770 cm^–1^ to the presence of Ni(O–Ti)_5_ units (calculated Raman shift: 733 cm^–1^). Finally, we assign the weak band at 841 cm^–1^ to the presence of Ti(O–Ti)_5_ units (calculated Raman shift: 853 cm^–1^). Note that since our samples have fairly large grains, we do not expect the grain boundaries themselves to contribute substantially to the Raman spectrum.^110,111^

Based on the spectra, we conclude that nearly all oxygen vacancies in our as-synthesized STN06 pellet are coordinated to Ni cations. This is consistent with previous computational work, which predicted that such associates would dominate at lower temperatures,^112^ and our lattice parameter measurements. Furthermore, the strong Ni(O–Ti)_4_ band and the weak Ni(O–Ti)_5_ band suggests considerable populations of Ni(O–Ti)_4_ units, a sign of heterogeneous distribution of Ni^2+^. We suspect that this peak is primarily associated with the Ni-rich regions that may form at grain boundaries.

Upon heating, STN06’s Raman spectra significantly changed. Our computational results indicate that these changes are linked to dissociation of oxygen vacancies from the Ni cations. At 300 °C, the peak at 537 cm^–1^ faded rapidly while the relative intensity of the Ni(O–Ti)_4_ band at 677 cm^–1^ decreased. Based on literature reports,^113−115^ we believe the peak at 537 cm^–1^ arises from STO’s TO_4_ fundamental mode, made Raman active by the presence of Ni(O–Ti)_4_ structural units, such as those present in (Ni_Ti_^″^–V_O_^••^–Ni_Ti_^″^–V_O_^••^)^×^. One possibility is that the TO_4_ mode is activated by a change in the long-range structure within the Ni-enriched regions. For example, there may be substantial B-site octahedral tilting when oxygen vacancies are present within these regions, as observed in the supercell containing (Ni_Ti_^″^–V_O_^••^–Ni_Ti_^″^–V_O_^••^)^×^. Meanwhile, a more-defined Ti(O–Ti)_5_ band (841 cm^–1^) appeared, and the weak Ni(O–Ti)_5_ band was no longer prominent. This corresponds to full dissociation of oxygen vacancies from the Ni cations.

At 400 °C, the relative intensity of the Ti(O–Ti)_5_ band continued to increase while the Ni(O–Ti)_4_ bands weakened. Furthermore, a more-defined band around 791 cm^–1^ began to take shape. Although literature reports often assign bands within this region to STO’s fundamental LO_4_ mode, we identify this Raman band as the local vibration of Ni(O–Ti)_6_ structural units based on our computational results (calculated Raman shift: 778 cm^–1^). As an additional check, we examined a reference spectrum for La_0.1_Sr_0.9_Ti_0.94_Ni_0.06_O_3-δ_ (LSTN). In LSTN, 10 mol % substitution of La^3+^ is introduced onto the Sr^2+^ site to partially charge compensate the 6 mol % Ni^2+^ substitution. This reduces the concentration of oxygen vacancies in the material, and therefore LSTN should have a high population of Ni(O–Ti)_6_ structural units. Indeed, we observed a peak at 804 cm^–1^, supporting our assignment for this peak.

At 600 °C, the Ni(O–Ti)_6_ and Ti(O–Ti)_5_ bands grew more intense while the Ni(O–Ti)_4_ band continued to weaken. The Ni(O–Ti)_5_ band, meanwhile, remained very weak and difficult to resolve. By 800 °C, the Ni(O–Ti)_6_ and Ti(O–Ti)_5_ features merged into a single broad band while other bands largely disappeared, indicating near-complete dissociation of (Ni_Ti_^″^–V_O_^••^)^×^ associates. After holding briefly at 800 °C, we cooled the STN06 shard and collected a final Raman spectrum. After cooling, the spectrum roughly resembled the starting spectra. The band at 537 cm^–1^ was considerably broader while the relative intensity of the band at 677 cm^–1^ was reduced, indicating lower populations of Ni(O–Ti)_4_ units. Compared to the initial spectrum, the Ni(O–Ti)_6_ and Ti(O–Ti)_5_ bands were slightly more prominent. These results indicated that the spectral changes were largely reversible, consistent with our assignments. Furthermore, this experiment suggested that Ni_Ti_^″^–V_O_^••^ reassociation could be at least partially hindered through quenching, which is a consequence of STN’s low oxygen ion conductivity at low temperatures.^80^

Our analysis of the temperature dependence of dominant defects aligns with the established mechanistic understanding that oppositely charged point defects form pairs at low temperatures, stabilized by mutual electrostatic interactions. Conversely, dissociation of these pairs into isolated defects takes place at higher temperatures. Rapid prediction of defect-induced Raman signatures allowed us to observe these events quickly and easily via Raman spectroscopy, without the need for intensive first-principles calculations or an extensive library of reference compounds and spectra.

### 3.3 Quenching-Induced Changes in Ni-Doped SrTiO_3_’s Raman Spectrum

Our methodology allowed us to assign point defects to individual features in STN06’s complex Raman spectrum and to observe the formation and dissociation of relevant point defect associates *in situ*. However, since high-temperature measurements led to significant peak broadening in Raman spectra, resolving the individual defect signatures was sometimes difficult. To better resolve the Raman shifts of the point defects, we carried out an additional quenching experiment to “freeze in” the relevant point defects and measure their Raman signatures at room temperature.

For this experiment, we selected a different STN06 shard that contained parts of the original pellet’s surface. We carried out the quenching experiment by heating this new STN06 shard in a furnace at 900 °C under a continuous flow of synthetic air. We held the sample at 10 h at 900 °C to give ample time for (Ni_Ti_^″^–V_O_^••^)^×^ disassociation then quenched it to room temperature by removing the sample directly from the furnace. The sample took roughly 1 min to cool from 900 °C to room temperature, after which we collected a postquench Raman spectrum. To evaluate the permanence of changes of Raman spectrum after quenching, the Raman spectrum of the quenched sample was measured again after 1 month of storage under ambient conditions.

**Figure 5:**
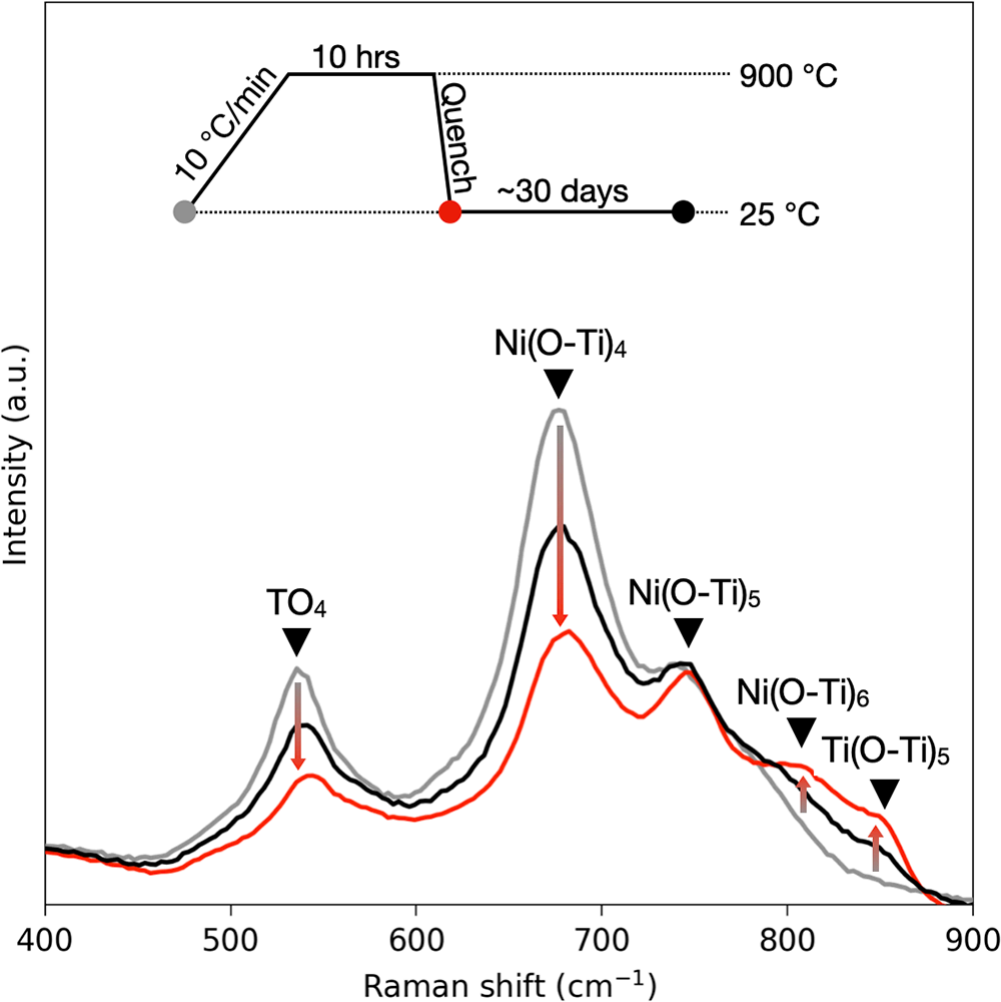
*Ex situ* Raman spectra of Sr(Ti_0.94_Ni_0.06_)O_3-δ_ measured at room temperature over the course of a quenching experiment.

The collected Raman spectra are shown in Figure 5. Before heating, STN06’s Raman spectrum exhibited Ni(O–Ti)_4_ bands (537 and 677 cm^–1^) and Ni(O–Ti)_5_ bands (739 cm^–1^). These bands were more prominent than those in the STN06 shard used in the previous *in situ* heating experiment, indicating differences in the distribution of Ni and oxygen vacancies between the interior and the surface of the sintered STN06 pellet. Such differences are well-established features of perovskite solid solution pellets.^116^ As before, the as-synthesized spectrum indicated a high degree of Ni_Ti_^″^–V_O_^••^ association (an intense Ni(O–Ti)_4_ band). However, the Ni(O–Ti)_5_ band was more intense than observed in our *in situ* experiment, suggesting a higher population of these structures closer to the pellet surface and allowing us to better resolve the Raman shift of this particular point defect.

After quenching, the Ni(O–Ti)_4_ bands weakened, as before. Alongside these changes, prominent Ni(O–Ti)_6_ and Ti(O–Ti)_5_ bands appeared (805 and 853 cm^–1^). Interestingly, the Ni(O–Ti)_5_ peak did not appreciably change, suggesting that the rapid quenching was insufficient to prevent recombination of single oxygen vacancies with isolated Ni cations. We rationalize this as follows. At high temperatures, oxygen vacancies are more-or-less uniformly distributed within our sample. Upon cooling, isolated Ni_Ti_^″^’s will likely have a single V_O_^••^ available nearby to trap; our quenching was not rapid enough to prevent this recombination. However, Ni-rich regions, from which the Ni(O–Ti)_4_ band arises, must collect oxygen vacancies from a comparably large region of the material to fully associate all contained Ni atoms. Although reassociation is apparently quite rapid, our quenching rates were sufficient to partially inhibit this process.

To test the permanence of the changes in the Raman spectra, a final Raman spectrum was measured after 30 days of storage under ambient conditions. After the 30 days, all features remained visible. However, the relative intensities of the bands at 542 and 678 increased while the intensities of the bands at 802 and 851 cm^–1^ weakened considerably. This indicates that, even at lower temperatures, diffusion of O^2–^ allows Ni_Ti_^″^/V_O_^••^ reassociation to continue.

In this experiment, measurements at room temperature allowed more reliable identification of the Raman shifts of the defect bands. Table 1 summarizes our measured and calculated Raman shifts associated with various ionic point defects in STN06. Despite the concessions made to reduce the computational cost of our calculations and despite the level of theory employed in our DFT calculations, our methodology was able to assign the experimental Raman shift to Ti(O–Ti)_5_ and Ni(O–Ti)_5_ structural units within 7 cm^–1^. Such a close match makes sense within our conception of STN06’s structure, as these structural units are found in isolation within the grain bulk.

**Table 1:** Raman Shifts of Measured and Calculated Raman Bands of STN06 at Room Temperature

experimental (cm^–1^)	computed (cm^–1^)	assigned structural unit
677	703	Ni(O–Ti)_4_
739	733	Ni(O–Ti)_5_
805	778	Ni(O–Ti)_6_
853	853	Ti(O–Ti)_5_

On the other hand, our calculations overestimated the Raman shift of the Ni(O–Ti)_4_ band by 26 cm^–1^ and underestimated the Raman shift of the Ni(O–Ti)_6_ band by 27 cm^–1^. These discrepancies may certainly be inherent to our DFT calculations, the isolated defect approach, or the harmonic approximation.^55^ However, our experimental observations give us confidence in our assignments, and we believe that the finite supercell size is responsible for most of the discrepancies. Substantial populations of Ni(O–Ti)_4_ units are largely present in Ni-rich regions which may deviate substantially from the isolated defect models. Similarly, since our STN06 sample’s Ni(O–Ti)_4_ bands are more intense than the Ni(O–Ti)_5_ bands, we believe Ni(O–Ti)_6_ units within the Ni-rich regions are the major contributor to the Ni(O–Ti)_6_ band that appears upon heating and/or quenching (as opposed to isolated Ni(O–Ti)_6_ units). Despite this, the supercell approach performed remarkably well, reducing the cost of our calculations and greatly easing analysis of our results.

## 4 Discussion

The above findings show that, armed with our computational approach, one can accurately assign features in the Raman spectra of solid-state ion conductors to predominant ionic point defects. The key features in our approach are its relatively low computational cost and its ability to isolate point defect signatures in a straightforward and intuitive way. Both features make it much easier to carry out joint computational-experimental Raman studies.

Our findings and methods are part of a field-wide move toward joint computational/experimental approaches. These approaches promise to accelerate material discovery for critical applications, such as renewable energy, while also uncovering novel and surprising insights into material phenomena. Within this context, we believe that the speed, flexibility, and convenience of Raman spectroscopy make it well-suited for high-throughput experimental studies on solid-state ion conductors. Synergizing high-throughput experiments with our computational approach offers a promising direction in the emerging area of data-driven materials science and engineering.^117^

It is also worthwhile to discuss our method within the context of more traditional computational study of point defects. Such studies typically rely on DFT-derived point defect formation energies^59^—calculated based on the average, 0 K structure—which are then used to rationalize various empirical observations. These calculations are a very powerful tool to study defect chemistry. However, reliable results are exceedingly difficult to obtain when studying solid-state ion conductors, whose properties are strongly influenced by dynamic processes occurring in regions of the potential energy surface which lie well away the idealized, 0 K structure.^118^ Even when correspondence between calculations and experiment is obtained, the accuracy of calculations can be difficult to gauge due to issues of measuring defect formation energies experimentally. Our approach, although based on idealized 0 K defect structures, overcomes these limitations by explicitly considering the dynamics of the system (subject to the limits of the harmonic approximation) as well as focusing on Raman spectra, which can be directly compared to easily collected experimental data. Furthermore, we have demonstrated that our approach produces results that are relevant to moderate- and high-temperature properties of solid-state ion conductors, all without needing to carry out expensive molecular dynamics simulations.^119^

We also emphasize that our approach is not limited to STN, which we have treated as a model system. Rather, we expect that it can be adapted relatively easily to study a wide range of defective materials. We foresee particularly interesting spectroscopic investigations of complex and relevant material systems such as garnets,^120,121^ argyrodites,^122,123^ or even high entropy solid-state ion conductors.^124,125^ All these materials require enormous simulation cells, for which assessment of defect Raman signatures with conventional first-principles calculations quickly exceeds reasonable computational time scales. Together with experimental Raman spectroscopy, our approach will substantially reduce the cost of identifying relevant defect signatures in solid-state ion conductors and, ultimately, help translate Raman observations into mechanistic insights and engineering principles.

Finally, we note that while our current method still relies on DFPT for calculating atomic Raman tensors, developments in ML continue to reduce the cost of Raman spectra calculations.^68,126−128^ Although these ML methods are still rather new, rapid progress in this area may soon offer additional low-cost routines for predicting atomic Raman tensors relevant to point defects. Incorporating ML Raman tensor predictions into our computational framework promises to further reduce computational cost. On a grander scale, we envision that such developments will drive the discovery of next-generation solid-state ion conductors.

## 5 Conclusions

Raman spectroscopy is a promising tool for characterizing ionic point defects in solid-state ion conductors for electro- and photochemical devices. Although Raman spectroscopy is considerably more straightforward and accessible than conventional point defect characterization methods, correct assignment of point defects to their Raman signatures requires time-consuming experiments or expensive calculations. In this work we developed a methodology to efficiently calculate the Raman signatures of ionic point defects that uses ML-accelerated frozen phonon calculations and “atomic Raman tensors” to evaluate polarizability directional derivatives with respect to individual atomic displacements. Our approach allows straightforward isolation and analysis of point-defect Raman signatures and, critically, fully exploits global symmetries and similarities between local atomic environments. Through this, reasonably accurate point defect Raman signatures can be derived at relatively low computational cost.

To demonstrate our method’s capabilities, we used it to analyze experimental Raman spectra of a model oxygen ion conductor: Ni-doped SrTiO_3_ (STN). Based on our calculations and experiments, we confirmed that the substitution of Ni^2+^ on the Ti^4+^ site of SrTiO_3_, a cubic perovskite with no first-order Raman activity, gives rise to Ni substitutional defects compensated by oxygen vacancies. We also resolved both the association of oxygen vacancies with Ni substitutional defects and the inhomogeneous distribution of Ni in STN, in doing so precisely assigning Raman peaks to the vibrations of Ti(O–Ti)_5_ and Ni(O–Ti)_*x*_ structural units.

The power of the joint computational-experimental approach enabled by our method becomes most apparent when we precisely determine changes in the defect chemistry within STN at varying temperatures. We found a predominant formation of Ni_Ti_^″^–V_O_^••^ pairs at low temperature and their dissociation into isolated Ni_Ti_^″^ and V_O_^••^ point defects at increased temperatures. Furthermore, we found a substantial number of aggregates with multiple Ni_Ti_^″^–V_O_^••^ associates at low temperatures, indicating the presence of Ni enriched domains. These mechanistic insights may prove critical to tailoring the migration pathways of oxygen ions via oxygen vacancies, whose presence as isolated species is preferred to allow efficient migration of oxygen ions. Furthermore, our method provides a facile way to screen different dopants and could be used, for example, to determine compositions with lower dopant/oxygen vacancy dissociation temperatures.

Compared to conventional frozen-phonon approaches, using our method reduced the computational cost of our calculations by roughly 80%. We expect that ongoing developments in the context of ML for predicting polarizability and Raman tensors may further accelerate our computational method. By enabling accurate simulation of ionic point defect Raman signatures at relatively low computational cost, this work is an important step in the development of Raman spectroscopy as a tool to characterize the nature of mobile and immobile point defects central to solid-state ion conductors and other complex functional materials.

## Supporting Information Available

Experimental and computational details, including literature survey, calculation details, benchmarking, and supplementary discussions (PDF)
