# Supplementary material for: Rapid Characterization of Point Defects in Solid-State Ion Conductors Using Raman Spectroscopy, Machine-Learning Force Fields, and Atomic Raman Tensors
Source: J Am Chem Soc. 2024 Sep 18;146(39):26863–76. doi: 10.1021/jacs.4c07812 (PMC11450927; doi:10.1021/jacs.4c07812)
Supplement: Supplementary file 1 — ja4c07812_si_001.pdf [file ja4c07812_si_001.pdf]

Supporting Information

Rapid Characterization of Point Defects in Solid-State Ion Conductors using Raman Spectroscopy, Machine-Learning Force Fields and Atomic Raman Tensors

Willis O'Leary [a], Manuel Grumet [b], Waldemar Kaiser [b], Tomáš Bučko[cd], Jennifer L. M. Rupp [ae]*, David A. Egger [bf]*

[a] Department of Materials Science and Engineering, Massachusetts Institute of Technology, Cambridge, MA, USA

[b] Department of Physics, TUM School of Natural Sciences, Technical University of Munich, Garching, Germany

[c] Department of Physical and Theoretical Chemistry, Faculty of Natural Sciences, Comenius University Bratislava, SK-84215 Bratislava, Slovakia

[d] Institute of Inorganic Chemistry, Slovak Academy of Sciences, SK-84236 Bratislava, Slovakia

[e] Department of Chemistry, TUM School of Natural Sciences, Technical University of Munich, Garching, Germany

[f] Atomistic Modeling Center, Munich Data Science Institute, Technical University of Munich, 85748 Garching, Germany

Corresponding authors:
*jrupp@tum.de
*david.egger@tum.de

Contents:

S.1 Previously reported Raman spectra for defective $SrTiO_3$ 2
S.2 Calculation details 4
S.3 Method details 5
S.3.1 Generation of machine-learning force fields 5
S.3.2 Calculation of polarizability derivatives in the atomic coordinate basis 5
S.3.3 Calculation of per-phonon Raman intensities 6
S.4 Performance of machine-learning force field frozen phonon calculations 8
S.5 DFT convergence tests 10
S.5.1 Frozen phonon calculations 10
S.5.2 Dielectric tensor calculations 11
S.6 Inheritance of atomic Raman tensors 13
S.7 Influence of supercell size on calculated Raman spectra 15
S.8 Discussion of computational cost 16
S.9 Experimental Details 17
S.10 Influence of Isolated Defects on Lattice Parameters 18

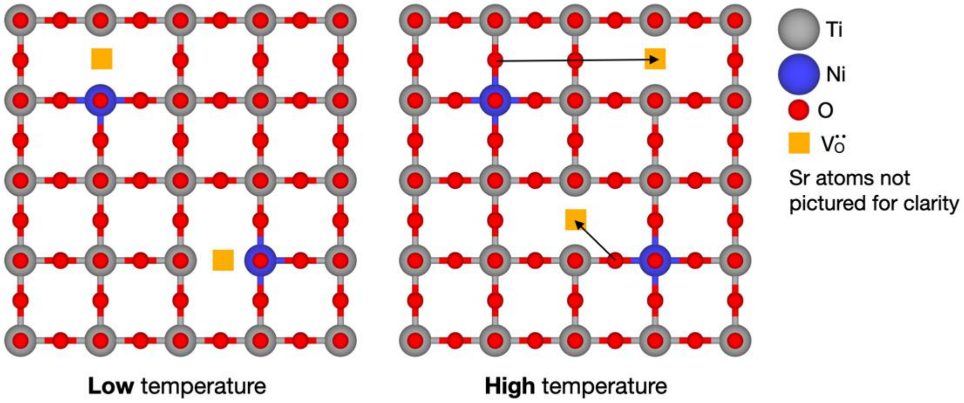

Figure S1. Schematic showing expected distribution of oxygen vacancies in Ni-substituted $SrTiO_3$ at low and high temperatures. Oxygen vacancies preferentially associate with Ni substitutional defects at low temperatures and dissociate at high temperatures.

S.1 Previously reported Raman spectra for defective $SrTiO_3$

Table S1. Experimentally observed strong Raman bands for $SrTiO_3$ materials

Ref.	Material	Raman Shift (cm^{-1})					Symmetry[a]	Cause[a]
[1]	$(Sr_{1-x}Ca_x)TiO_{3-\delta}$	547					TO_4	Phase transition
[2]	$Sr(Ti_{1-x}Mg_x)O_{3-\delta}$	545					TO_4	Mg substitution
				762			Local	Octahedral breathing
					795		LO_4	Mg substitution
[3]	Reduced $SrTiO_{3-\delta}$	550					TO_4	Oxygen vacancies, Ti^{3+}
			630					Oxygen vacancies, Ti^{3+}
			700					Oxygen vacancies, Ti^{3+}
					810		LO_4	Oxygen vacancies, Ti^{3+}
[4]	$Sr(Ti_{1-x}Fe_x)O3_{-\delta}$	545					TO_4	Symmetry breaking
			690				Local	$Fe^{4+}O_6$ (Jahn-teller)
					795		LO_4	Symmetry breaking
[5]	$SrTiO_{3-\delta}$	545						Impurities or oxygen vacancies
					795			Impurities or oxygen vacancies
[6]	$SrTiO_{3-\delta}$	550					TO_4	Sr/Ti nonstoichiometry
					800		LO_4	Sr/Ti nonstoichiometry
[7]	$Sr(Ti_{0.9}Ni_{0.1})O_{3-\delta}$	545					TO_4	Ni, oxygen vacancies
			700				Local	Ni, oxygen vacancies
					793		LO_4	Ni, oxygen vacancies
[8]	$Sr(Ti_{1-x}Co_x)O_{3-\delta}$	~550					TO_4	
			690					Oxygen vacancies or Co
					~800		LO_4	
[9]	Reduced $SrTiO_{3-\delta}$	549						Oxygen vacancies
						860		Oxygen vacancies
[10]	$Sr(Ti_{0.7}Fe_{0.3})O_{3-\delta}$	550					TO_4	Defects
			695					$Fe^{4+}O_6$
					~800		LO_4	Defects
[11]	$(Sr_{0.96}Zr_{0.04})(Ti_{0.90}Ni_{0.1})O_{3-\delta}$	547					TO_4	Defects
			641				TO_1+TO_4	Defects
			702				LA+LO_3	Defects
					797		LO_4	Defects

[12]	$(Sr_{1-x}Pr_x)TiO_{3-\delta}$	520					TO_4	Pr doping
					~800		LO_4	Pr doping
This	$Sr(Ti_{0.94}Ni_{0.06})O_{3-\delta}$	537					TO_4	$Ni(O\text{-}Ti)_4$ (Ni-enriched regions)
work			677				Local	$Ni(O\text{-}Ti)_4$ (Ni-enriched regions)
				739			Local	$Ni(O\text{-}Ti)_5$
					805		Local	$Ni(O\text{-}Ti)_6$
						853	Local	$Ti(O\text{-}Ti)_5$

[a] If assigned.

Table S1 reviews literature reports on the appearance of strong Raman bands above 500 cm^{-1} in $SrTiO_3$ (STO) materials that are absent in pure STO. Since STO possesses no first-order Raman bands, the appearance of strong bands is an indication of deviations from the perfect STO structure. This is often related to the intentional introduction of dopants on the perovskite A-site[1,12] or B-site[2,4,7,8] but can also be related to material processing, for example treatment under a reducing atmosphere[3,9]. The most common activated Raman band lies around 550 cm^{-1}, which is overwhelmingly assigned to the TO_4 fundamental vibration of STO. In this case, the presence of a wide variety of point defects (or in one case a phase transition[1]) appears to impart this mode with first-order Raman activity. A band around 800 cm^{-1} is also frequently observed; this is typically assigned to the LO_4 fundamental vibration of STO, again activated by the presence of defects.

Since STO's TO_4 and LO_4 fundamental vibrations can be activated by any number of point defects, these bands provide little information on the specific nature of the point defects present. Of more interest are new Raman modes between 600 and 800 cm^{-1}, as pure STO has no optical phonons within this frequency range[13]. Appearance of modes within this region, particularly around 700 cm^{-1}, have been reported[2–4,7,10,11]. Since these modes appear within the vibrational band gap, they are likely due to some sort of defect-specific local modes. These bands are a rich source of information on the point defects present, and their presence makes STO an ideal model system on which to apply the computational methodology presented in this work.

Several previous reports have attempted to predict the Raman signatures of point defects in STO using DFT calculations. Rusevich *et al.* performed static Raman spectrum calculations on neutral oxygen vacancies in a $2 \times 2 \times 2$ STO supercell. They found that the relative spin polarizations of the charge-compensating Ti^{3+} ions had a major impact on the Raman spectra. However, a $2 \times 2 \times 2$ model might be too small to compare to real materials[14]. Krasnenko *et al.* treated the Raman spectrum of $SrTiO_3$ surfaces, finding that the surfaces resulted in several new

Raman modes[15]. Overall, computational literature studying vibrational signatures of point defects in STO is sparse.

S.2 Calculation details

DFT calculations were performed according to the generalized gradient approximation using the Perdew–Burke–Ernzerhof (PBE)[16] functional as implemented in the Vienna Ab initio Simulation Package (VASP) v.6.3.0[17–20]. A 400 eV plane-wave cutoff was used in all calculations unless stated otherwise. Calculations on Ni-containing structures were spin-polarized (to accommodate Ni^{2+}), while all other calculations were non-spin-polarized. Electrons were added or removed to ensure Ni and Ti adopted 2+ and 4+ oxidation states respectively. The projector augmented-wave (PAW) method[20,21] was applied using the PAW pseudopotentials supplied with VASP (v.5.4). For Sr, the pseudopotential treating semi-core *s*-states as valence states was selected. For both Ti and Ni, pseudopotentials treating semi-core *p*-states as valence states were selected. The standard pseudopotential for O was used. To improve treatment of long-range forces, the DFT-D3 method with Becke-Johnson damping was applied[22]. To allow for more realistic charge localization on the B-site cations, +U corrections were included[23]; U parameters of U_{Ti}=3 eV U_{Ni}=6 eV were used. These lie within previously suggested ranges[20,30–3224,25] and are consistent with our previous study[26]. Partial occupancy smearing was done using Gaussian smearing (0.1 eV width).

We constructed cubic STO supercells using lattice parameters for pure STO, as determined by DFT geometry optimization (a = 3.940 Å). In all calculations, the lattice parameters were kept fixed unless noted otherwise. $4 \times 4 \times 4$ and $5 \times 5 \times 5$ STO supercells were treated considering only the Γ-point. $3 \times 3 \times 3$ supercells were treated with a $2 \times 2 \times 2$ Monkhorst *k*-point grid[27]. All force calculations were done using VASP's accurate precision-mode. Structural relaxations were performed using a per-atom force convergence threshold of 10^{-2} eV/Å and an electronic convergence threshold of 10^{-6} eV. Γ-point harmonic modes were determined with the frozen phonon approach using finite differences. These calculations were performed within VASP using a step size of 0.015 Å. Dielectric tensors (or equivalently, polarizability tensors) were calculated using density functional perturbation theory[28,29] (DFPT) and an electronic convergence cutoff of 10^{-5} eV using VASP's normal precision-mode. To improve convergence of the dielectric tensors with respect to the number of *k*-points, the derivatives of the cell-periodic part of the orbitals were

treated using finite differences. We chose the above parameters based on benchmarks presented in **Section S.5.**

S.3 Method details

S.3.1 Generation of machine-learning force fields

We generated a machine-learning force field (MLFF) for each structure considered. For each given structure, we first constructed a 20-structure training set using the Monte Carlo "rattle" approach implemented in the Hiphive package[30]. In this approach, training structures are generated by perturbing all atomic positions from their lowest-energy positions, generating a "rattled" structure that superficially resembles a molecular dynamics snapshot. When generating these structures, we used a rattle amplitude of 0.015 Å and a minimum distance parameter (which defines the Monte Carlo probabilities) of 1.75 Å.

We calculated the atomic forces for all structures in the training set, then used these forces to train a MLFF. We used the MLFF implementation in VASP[17–20]; details on this particular MLFF can be found in refs. 31–33. MLFF training was done in two steps. First, we selected basis sets using strict training parameters (radial descriptor gaussian width of 0.3 Å with 12 radial basis functions). We then refitted using singular value decomposition and VASP's default MLFF parameters (radial descriptor gaussian width of 0.5 Å with 8 radial basis functions). To ensure accurate prediction of forces, training weights on the forces, energies, and stresses were fixed at 20, 1, and 1 respectively during all training phases.

We found that the cutoff radii for the radial and angular descriptors making up the MLFF needed to be adjusted depending on the size of the system to obtain good results. For $3 \times 3 \times 3$ STO supercells, we used 8 Å and 5 Å cutoffs for the radial and angular descriptors respectively. For larger $4 \times 4 \times 4$ and $5 \times 5 \times 5$ STO supercells, we found larger cutoffs were necessary to maintain accuracy; here, we used 12 Å and 8 Å cutoffs for the radial and angular descriptors respectively.

S.3.2 Calculation of polarizability derivatives in the atomic coordinate basis

Conventionally, the polarizability derivatives with respect to normal coordinates $\partial\alpha_{\mu\nu}/\partial Q_p$ for every phonon mode p are computed directly with central differences. This requires generating configurations with atoms displaced along each phonon eigendisplacement Q_p. Calculating $\alpha_{\mu\nu}$ from first principles can be done *via* DFPT. However, doing this for each of these

configurations would be computationally very expensive. We note that the directional derivative $\partial\alpha_{\mu\nu}/\partial Q_p$ is readily computed using the gradient:

$$\frac{\partial\alpha_{\mu\nu}}{\partial Q_p} = \sum_{i=1}^{N_{at}} \sum_{\lambda=x,y,z} \left.\frac{\partial\alpha_{\mu\nu}}{\partial x_{i,\lambda}}\right|_{x_0} \cdot \frac{\partial x_{i,\lambda}}{\partial Q_p} = \sum_{i=1}^{N_{at}} \boldsymbol{\nabla}_i \alpha_{\mu\nu}(\boldsymbol{x}_0) \cdot \boldsymbol{Q}_{p,i} \qquad (S1)$$

where $\boldsymbol{x}_0$ defines the equilibrium structure of the material. By using Equation S1, we can compute $\partial\alpha_{\mu\nu}/\partial Q_p$ in the atomic coordinate basis. The so-called atomic Raman tensors $\partial\alpha_{\mu\nu}/\partial x_{i,\lambda}$ are calculated *via* central differences. This requires calculating the polarizabilities of two supercells in which atom i has been displaced slightly in the $\pm\lambda = x, y, z$ directions. We chose a displacement size of 0.16 and 0.26 Å for displacements parallel and orthogonal to the B-O bonds in the perovskite. These values correspond to the scale of typical displacements due to thermal motion.

Using Equation S2 to compute the $\partial\alpha_{\mu\nu}/\partial Q_p$'s of a system has several advantages. First and foremost, the derivatives $\partial\alpha_{\mu\nu}/\partial x_{i,\lambda}$ need only be calculated for the crystallographically distinct sites. On a single site, the derivatives $\partial\alpha_{\mu\nu}/\partial x_{i,\lambda}$ need only be determined for directions which are crystallographically equivalent. For example, if displacements of atom 3 in the x and y directions result in crystallographically equivalent structures, then $\partial\alpha_{\mu\nu}/\partial x_{3,y}$ can be obtained by applying the symmetry operation (a rotation, in this case) to $\partial\alpha_{\mu\nu}/\partial x_{3,y}$. Furthermore, as mentioned in the main text, evaluation in the atomic coordinate basis makes it very straightforward to focus on the contribution of specific atomic species. For example, if only the contribution of the oxygen atoms is of interest, then the $\partial\alpha_{\mu\nu}/\partial x_{i,\lambda}$ terms for the other atoms do not need to be calculated.

S.3.3 Calculation of per-phonon Raman intensities

We calculated Raman spectra assuming a backscattered illumination/observation geometry and a randomly oriented polycrystalline sample. These conditions were consistent with our experimental measurements. Under these conditions, the Raman intensity at Raman shift v, $I(v)$, is calculated using the well-established formula[34,35]

$$I(v) \propto \frac{(v_{laser} - v)^4}{v} \frac{1}{1 - e^{\frac{-hcv}{kT}}} (45a^2 + 7\gamma^2) \qquad (S3)$$

This expression depends on v_{laser}, the wavenumber of the illuminating laser, and includes a quantum mechanical correction factor. The key material-dependent quantities are a^2 and γ^2, two invariant quantities derived from the polarizability tensor derivatives. They are defined as

$$a^2 = \frac{1}{9}\left(\frac{\partial \alpha_{xx}}{\partial Q_p} + \frac{\partial \alpha_{yy}}{\partial Q_p} + \frac{\partial \alpha_{zz}}{\partial Q_p}\right)^2 \tag{S4}$$

and

$$\gamma^2 = \frac{1}{2}\left(\left(\frac{\partial \alpha_{xx}}{\partial Q_p} - \frac{\partial \alpha_{yy}}{\partial Q_p}\right)^2 + \left(\frac{\partial \alpha_{xx}}{\partial Q_p} - \frac{\partial \alpha_{zz}}{\partial Q_p}\right)^2 + \left(\frac{\partial \alpha_{yy}}{\partial Q_p} - \frac{\partial \alpha_{zz}}{\partial Q_p}\right)^2\right) + 3\left(\left(\frac{\partial \alpha_{xy}}{\partial Q_p}\right)^2 + \left(\frac{\partial \alpha_{xz}}{\partial Q_p}\right)^2 + \left(\frac{\partial \alpha_{yz}}{\partial Q_p}\right)^2\right) \tag{S5}$$

S.4 Performance of machine-learning force field frozen phonon calculations

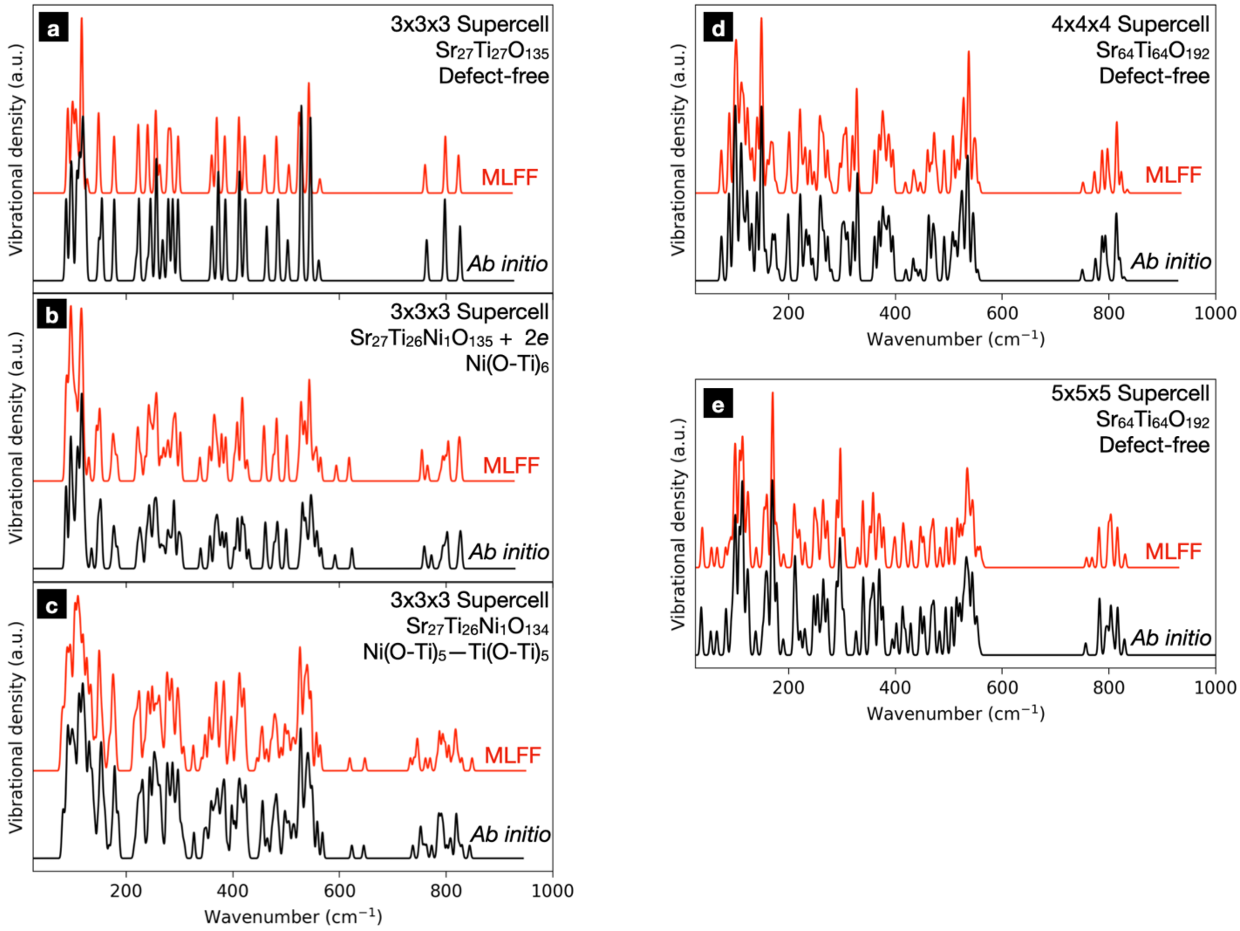

Figure S2. Phonon density of states for various test systems derived from frozen phonon calculations done using both DFT and our trained machine-learning force fields. The supercell size, stoichiometry, and relevant defective structural units are included in insets for each plot. To aid in visualization, discrete peaks were convoluted by Gaussians with standard deviation of 2 cm^{-1}. The MLFF adequately reproduced the *ab initio* results well for supercells of various sizes containing various point defects.

To test the accuracy of our MLFF-based frozen phonon calculations, we compared our calculated phonon density of states for various test systems against those derived from full DFT-level frozen phonon calculations. The results of these tests are shown in **Figure S2**. We began with a $3 \times 3 \times 3$ STO supercell and tested performance for the systems containing no point defects (**Figure S2a**), Ni_{Ti}'' (**Figure S2b**), and $(Ni_{Ti}'' - V_O^{\cdot\cdot})^x$ (**Figure S2c**)**.** The MLFF performed well in all three cases. In addition, we found that good performance was achieved on defect-free $4 \times 4 \times 4$ and $5 \times 5 \times 5$ STO supercells (**Figure S2d,e**). Overall, we are confident that our MLFF-based approach is effective at reproducing the results of *ab initio* frozen phonon calculations for all systems simulated in this work.

Table S2. Calculated versus experimental frequencies (cm^{-1}) of Γ-point phonons of $SrTiO_3$

DFT (PBE+U, 4 × 4 × 4 supercell)	MLFF (4 × 4 × 4 supercell)	Experiment (297 K)[36,37]
102	102	91
194	194	170
221	220	265
554	557	547

To further interrogate the accuracy of our calculations, we compared the calculated frequencies of STO's Γ-point phonons against those measured with inelastic neutron scattering[36,37]. The results of this comparison are presented in **Table S2.** As expected, the MLFF calculations were in excellent agreement with DFT-level calculations. We observed mostly satisfactory agreement between calculated frequencies and experiment, though the calculations underestimated the frequency of STO's 265 cm^{-1} phonon by 45 cm^{-1}. This level of disagreement was not surprising considering the relatively low level of theory employed (PBE functional, including a Hubbard +U parameter on Ti). Furthermore, we noted that STO's 265 cm^{-1} phonon involves motion of oxygen atoms perpendicular to the Ti-O-Ti bonds. Meanwhile, the high frequency phonons analyzed in this work involved motion of oxygen atoms *parallel* to the Ti-O-Ti bonds (i.e. breathing modes of BO_x motifs). STO's 547 cm^{-1} phonon has this character and is accurately treated by our methods, which gives us confidence in the calculated phonon frequencies relevant to this work.

S.5 DFT convergence tests

S.5.1 Frozen phonon calculations

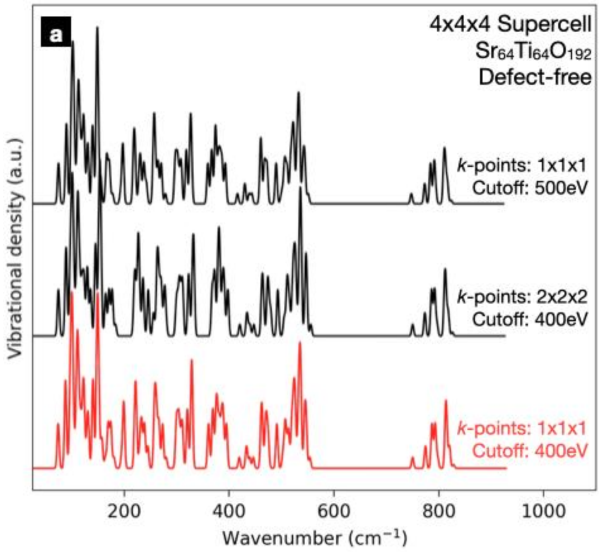

Figure S3. Phonon density of states for defect-free (a) $4 \times 4 \times 4$ and (b) $3 \times 3 \times 3$ STO supercells calculated *via* the frozen phonon method using various DFT parameters. To aid in visualization, discrete peaks were convoluted by Gaussians with standard deviation of 2 cm^{-1}. Curves and parameters in red are sufficient to obtain accurate vibrational information for these systems.

We carried out several benchmark calculations to ensure the DFT-level frozen phonon calculations presented in **Section S.4** were well-converged. **Figure S3a** shows the DFT-derived phonon density of states for a $4 \times 4 \times 4$, defect-free STO supercell calculated using various k-point densities and the plane wave energy cutoffs. We found that Γ-point calculations utilizing a 400 eV plane-wave cutoff were sufficiently accurate. Similarly, we found that the $3 \times 3 \times 3$, defect-free STO supercell could be accurately treated using a $2 \times 2 \times 2$ k-point grid and a 400 eV plane-wave cutoff (**Figure S3b**).

We used identical parameters when calculating the atomic forces in the "rattled" structures making up our MLFF training sets. Since the "rattled" structures are quite distorted compared to the minimum energy structures, the per-atom forces are larger than those encountered in frozen phonon calculations. Therefore, the strict parameters needed for frozen phonon calculations are more than sufficient for force calculations on these structures. It is possible that even less strict DFT parameters can be used here; this presents yet another avenue to reduce the computational cost of calculating vibrational properties.

S.5.2 Dielectric tensor calculations

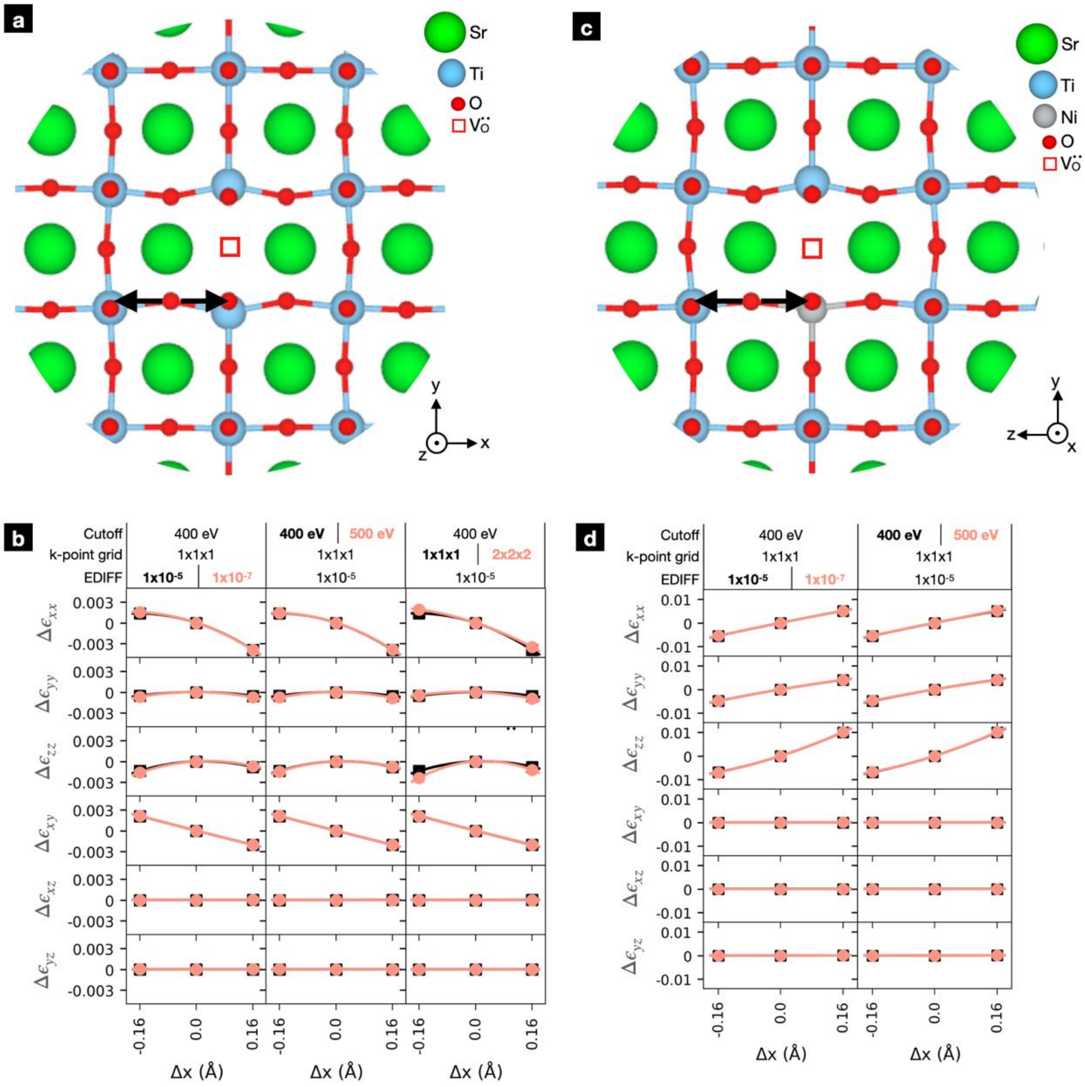

Figure S4. Dielectric tensor convergence tests. (a) Structure used to test a Ni-free system containing a single $V_O^{\cdot\cdot}$. Influence of displacement of the pictured oxygen on the dielectric tensor was tested. (b) Changes in the distinct components of the dielectric tensor as functions of oxygen displacement. (c) and (d) present analogous tests on a Ni-containing system (containing $(Ni_{Ti}'' - V_O^{\cdot\cdot})^x$).

We performed a handful of DFPT calculations with varying parameters to ensure that the dielectric tensor calculations in this work were accurate. We first performed tests on a Ni-free system: the $4 \times 4 \times 4$ STO supercell containing a $V_O^{\cdot\cdot}$ (and two charge-compensating holes). We displaced an oxygen close to the $V_O^{\cdot\cdot}$ ±0.16 Å along its bonding direction. Dielectric tensors were calculated for the reference and for the two displaced structures. We systematically varied the

parameters involved in the calculations, including the electronic convergence cutoff (1x10^{-5} eV vs. 1x10^{-7} eV), plane-wave energy cutoff (400 eV vs. 500 eV), and k-point grid ($1 \times 1 \times 1$ vs. $2 \times 2 \times 2$). The results of the convergence tests for all distinct components of the dielectric tensor are shown in **Figure S4b**. We found that calculations using a convergence cutoff of 1x10^{-5} eV, a plane-wave cutoff of 400 eV, and a $1 \times 1 \times 1$ k-point grid were well-converged for the Ni-free system. Next, we performed a similar convergence test on a Ni-containing system: the $4 \times 4 \times 4$ STO supercell containing $(Ni_{Ti}'' - V_O^{\cdot\cdot})^x$ (**Figure S4d**). A k-point test was not carried out due to memory constraints. The remaining results were similar.

Based on these tests, we used a convergence cutoff of 10^{-5} eV, a plane-wave cutoff of 400 eV, and a $1 \times 1 \times 1$ k-point grid in our dielectric tensor calculations on $4 \times 4 \times 4$ supercells. We noted that these parameters were identical to those needed for force calculations. Therefore, we calculated dielectric tensors for $3 \times 3 \times 3$ STO supercells using the same parameters used to calculate forces on these systems. Due to computational constraints, we did not calculate dielectric tensors on for the $5 \times 5 \times 5$ supercells, instead opting to use the atomic Raman tensors calculated on smaller systems to these larger systems as noted in **Supporting Information Section S.7**.

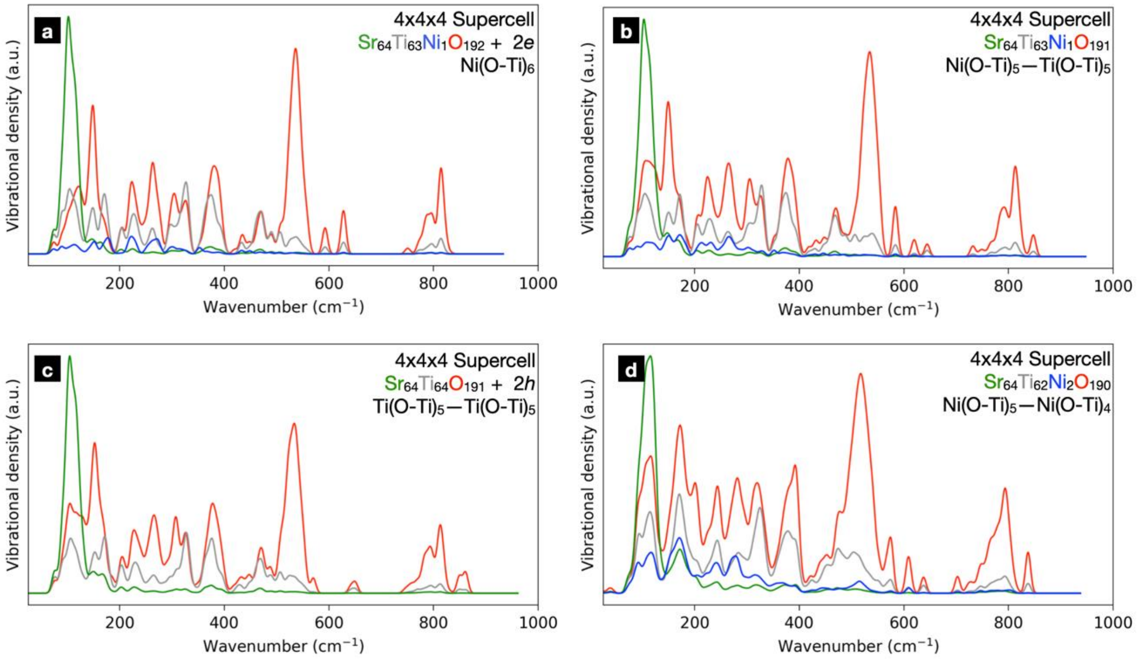

Figure S5. Element-projected partial phonon density of states for $4 \times 4 \times 4$ supercells containing various point-defects (see insets). To aid in visualization, discrete peaks were convoluted by Gaussians with standard deviation of 2 cm^{-1}. Above 600 cm^{-1}, harmonic modes are dominated by the movement of oxygen atoms.

S.6 Inheritance of atomic Raman tensors

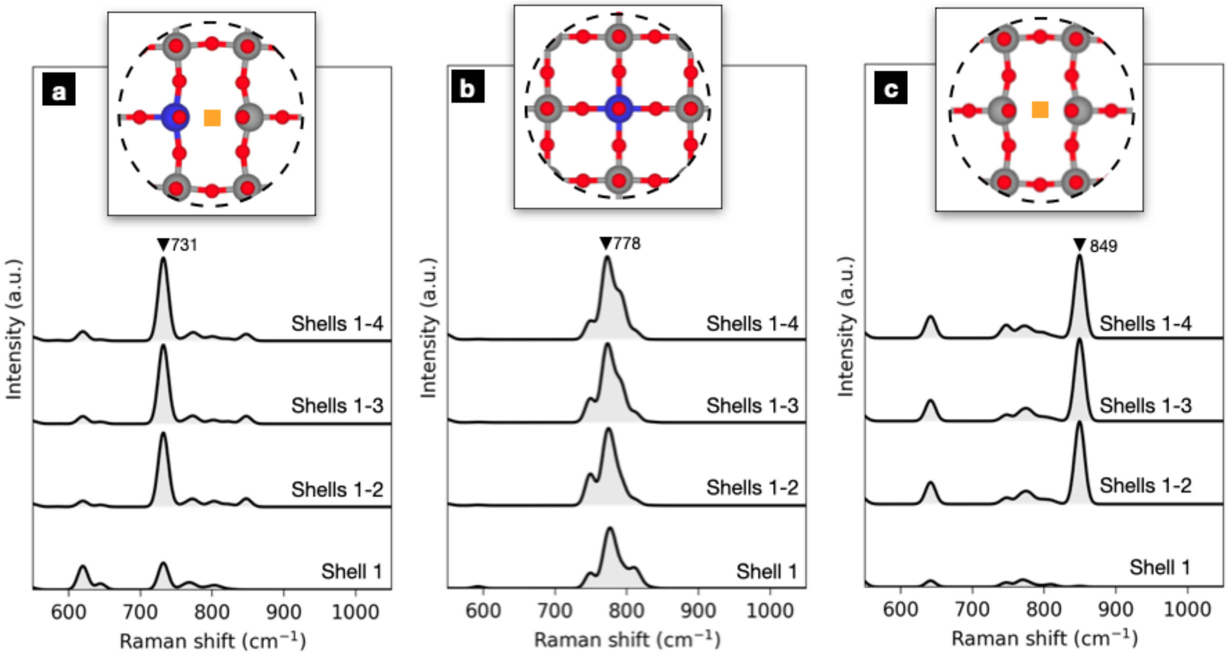

Figure S6. Structures and calculated partial Raman spectra of selected point defects considered in this study. Several partial Raman spectra are presented accounting for oxygens from a greater and greater number of nearest neighbor shells (based on pure STO). To obtain representative first-order Raman spectra, we found that we only needed to include oxygens coordinated to $Ti(O\text{-}Ti)_5$ and $Ni(O\text{-}Ti)_x$ structural units. To aid in visualization, discrete peaks were convoluted by Gaussians with standard deviation of 7 cm^{-1} is applied to all spectra.

As discussed in the main text, we focused on STN's Raman spectra above 600 cm^{-1}, as above this wavenumber phonons are dominated by movement of oxygen anions (**Figure S5**). By making this choice, we could estimate polarizability tensor derivatives above this wavenumber using only the atomic Raman tensors belonging to oxygen atoms. This drastically reduced the cost of our Raman calculations. Furthermore, we found that terms for only a handful of oxygen atoms needed to be calculated to obtain a nearly complete Raman spectra above 600 cm^{-1}. This result is presented in **Figure S6** in which we calculate the Raman spectra of various defective $4 \times 4 \times 4$ STO supercells accounting for oxygen atomic Raman tensors from a progressively larger number of nearest-neighbor-shells (defined in the context of the pure STO structure). In these tests, we consider three defects: Ni_{Ti}'' (**Figure S6a**), $(Ni_{Ti}'' - V_O^{\cdot\cdot})^x$ (**Figure S6b**), and $V_O^{\cdot\cdot}$ (**Figure S6c**).

The convergence of the Raman spectra with the inclusion of progressively larger number of nearest-neighbor shells is a natural consequence of decreasing structural distortions further

away from a point defect. This is illustrated in **Figure S7** for Ni_{Ti}'', $(Ni_{Ti}'' - V_O^{\cdot\cdot})^x$, and $V_O^{\cdot\cdot}$. Here, we plot the absolute atomic displacements of each atom in our relaxed structures (versus pure $SrTiO_3$) as a function of the distance of each atom from the respective point defect. Atomic displacements decrease asymptotically to zero far from the point defects, indicating that the local structure far away from each point defect closely resembles that of $SrTiO_3$. Therefore, atomic Raman tensors for atoms far away from point defects are close to those of $SrTiO_3$ (≈0). Therefore, the inclusion (or omission) of these atomic Raman tensors has little impact on the calculated Raman spectrum of the point defect in question.

For all defects, we found that we only needed to include atomic Raman tensors for oxygens contained within $Ti(O\text{-}Ti)_5$ and $Ni(O\text{-}Ti)_x$ structural units. This translated to inclusion of only the first nearest-neighbor shells for Ni_{Ti}'' and the first two nearest-neighbor shells for all other point defects. This result was expected, as STO's lack of first-order Raman activity implies that the oxygen atoms most significantly perturbed by a given point defect will contribute the most to that defect's first-order Raman signature. In this case, oxygens near a defect should be the most perturbed and therefore contribute most to the overall spectrum.

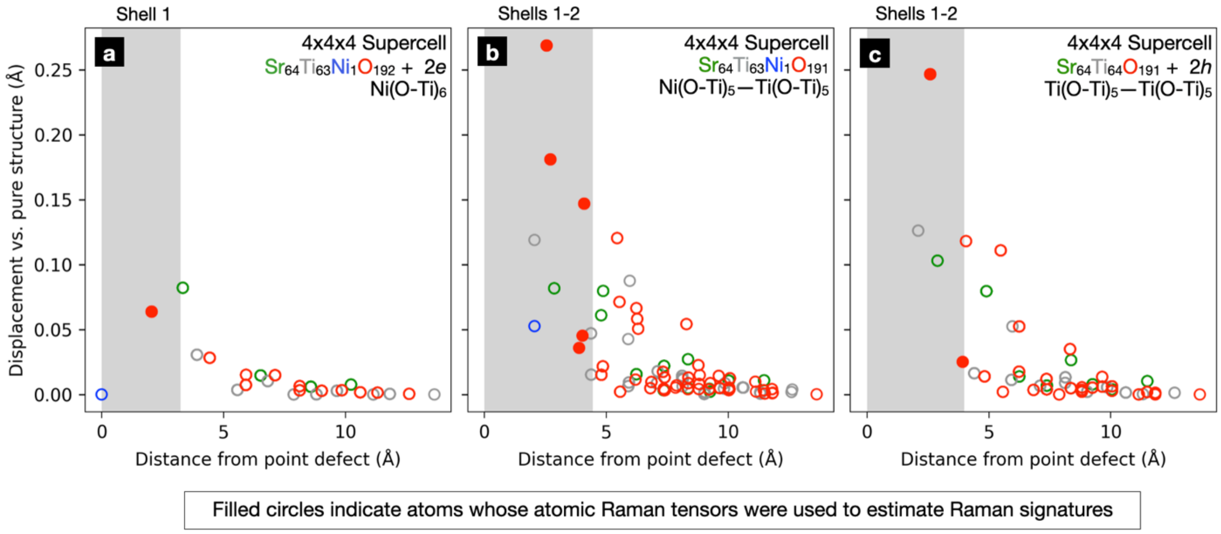

Figure S7. Absolute atomic displacements (versus pure $SrTiO_3$) of atoms as a function of distance from the point defect for selected point defects considered in this study. Atomic displacements decrease further from the defects, indicating that local atomic structure far from point defects closely resembles pure $SrTiO_3$. Points are filled according to the consideration of each atom's atomic Raman tensors when calculating polarizability derivatives. The distance interval of the nearest-neighbor shells used to construct the Raman spectra presented in the main text are shaded in gray.

S.7 Influence of supercell size on calculated Raman spectra

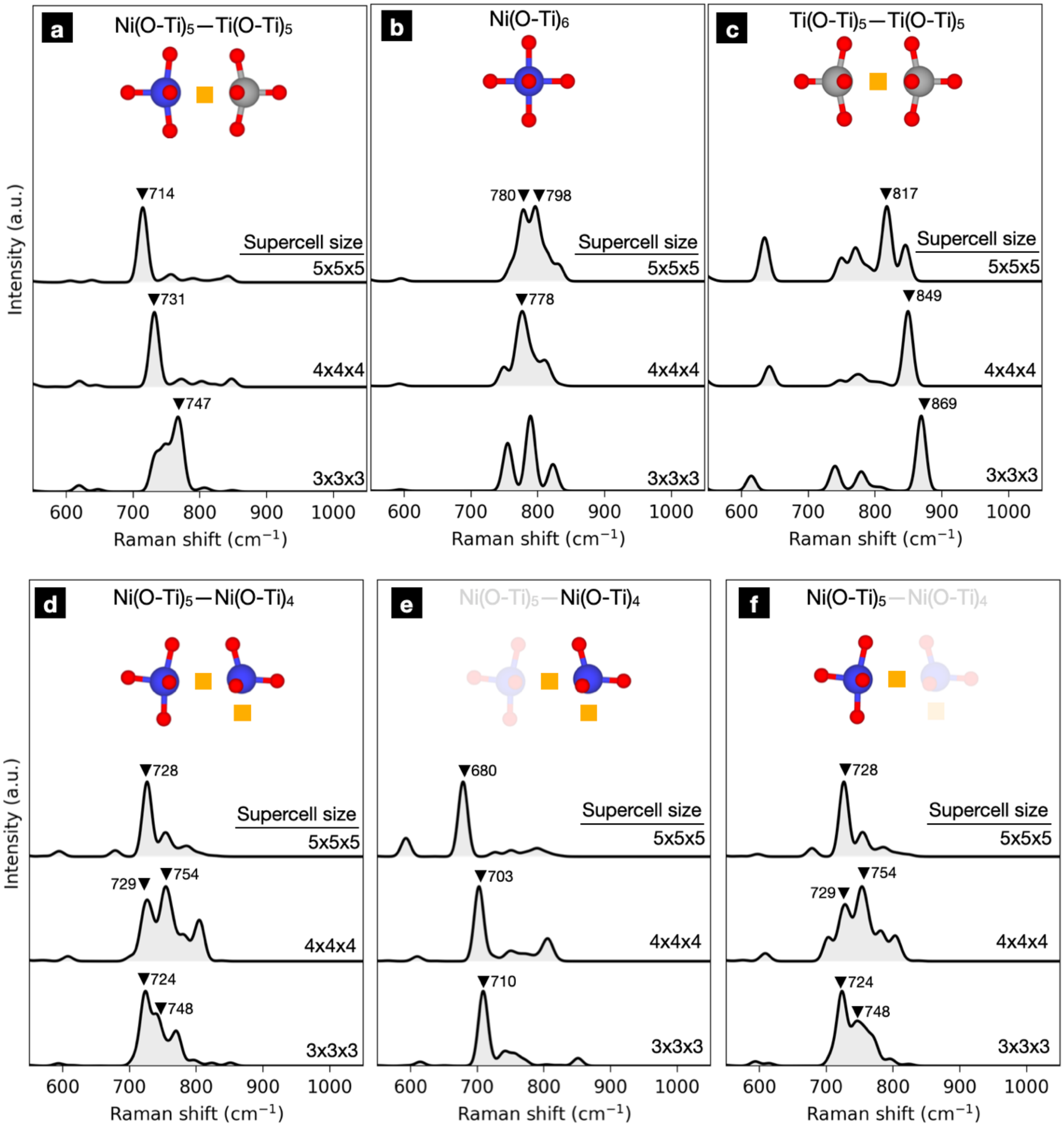

Figure S8. Raman signatures calculated using various sizes of supercells for (a) $(Ni_{Ti}'' - V_O^{\cdot\cdot})^x$, (b) Ni_{Ti}'', (c) $V_O^{\cdot\cdot}$, and (d) $(Ni_{Ti}'' - V_O^{\cdot\cdot} - Ni_{Ti}'' - V_O^{\cdot\cdot})^x$. Calculated signatures for the subunits of $(Ni_{Ti}'' - V_O^{\cdot\cdot} - Ni_{Ti}'' - V_O^{\cdot\cdot})^x$ are presented in (e) and (f). The position of the major band was dependent on the size of the supercell, but remained in similar ranges of Raman shifts relative to each other. To aid in visualization, discrete peaks were convoluted by Gaussians with standard deviation of 7 cm^{-1}.

As an additional benchmark, we compared the Raman spectra of all point defects considered in this study using $3 \times 3 \times 3$, $4 \times 4 \times 4$, and $5 \times 5 \times 5$ STO supercells. In these

calculations, only oxygen atomic Raman tensors from the Ti(O-Ti)$_5$ and Ni(O-Ti)$_x$ units were included. As in the main text, we exclude atomic Raman tensors for oxygens that are part of the Ti(O-Ti)$_5$ unit formed adjacent to $(Ni''_{Ti} - V_O^{\cdot\cdot} - Ni''_{Ti} - V_O^{\cdot\cdot})^x$, as these oxygens do not contribute meaningfully to the Raman spectra. The atomic Raman tensors were calculated explicitly for the $3 \times 3 \times 3$ and $4 \times 4 \times 4$ supercells. Due to computational constraints, the $4 \times 4 \times 4$ atomic Raman tensors were reused for the $5 \times 5 \times 5$ supercells. However, in the case of $(Ni''_{Ti} - V_O^{\cdot\cdot} - Ni''_{Ti} - V_O^{\cdot\cdot})^x$, the atomic Raman tensors of the $3 \times 3 \times 3$ supercell were reused; this was done because the structures of the $3 \times 3 \times 3$ and $5 \times 5 \times 5$ supercells were quite similar, while the structure of the $4 \times 4 \times 4$ supercell contained substantial octahedral tilting. This was ultimately a consequence of the odd number of unit cells contained within the $3 \times 3 \times 3$ and $5 \times 5 \times 5$ supercells, which prevented the octahedral tilting seen in the $4 \times 4 \times 4$ supercell.

The results of these calculations are shown in **Figure S8**. Although the shapes and positions of the defect-induced Raman bands were dependent on supercell size, the major peaks remained in similar wavenumber ranges, especially when considered in relation to the other point defects. Although this dependence may be in part due to interaction of the larger point defects with their periodic images, we believe that long-range lattice distortions induced by the defects are the primary cause. In an extreme case, $(Ni''_{Ti} - V_O^{\cdot\cdot} - Ni''_{Ti} - V_O^{\cdot\cdot})^x$ gave rise to long-range lattice distortions. We conclude that very large supercells (beyond $5 \times 5 \times 5$) may be required to truly isolate these point defects. The high computational cost of such simulations presents a significant barrier, but we are skeptical that such simulations are particularly instructive. Although the point defects will become properly isolated, the match between calculated and experimental Raman shifts is unlikely to improve in materials containing high concentrations of point defects.

S.8 Discussion of computational cost

We compare the computational cost of our method against that of the conventional, frozen-phonon-based method for Raman spectrum calculations in **Table S3**. Compared to frozen phonon calculations with Phonopy, our MLFF methodology dramatically reduced the number of DFT-level force calculations required to derive phonon frequencies and displacements. The speed-up became more dramatic in low-symmetry systems. Most importantly, our methodology's flexibility afforded several opportunities to greatly reduce the number of polarizability calculations needed to derive Raman intensities above 600 cm^{-1}. These opportunities are discussed in the main text.

Table S3. Computational cost of Raman spectrum calculations (>600 cm^{-1}) on $4 \times 4 \times 4$ STO supercells containing a single point defect or point defect associate

	Required force calculations		Required polarizability calculations	
Point Defect	Conventional	Our method	Conventional	Our method
Ni_{Ti}''	64	20	100	6
$V_O^{\cdot\cdot}$	157	20	65	10
$(Ni_{Ti}'' - V_O^{\cdot\cdot})^x$	310	20	78	20
$(Ni_{Ti}'' - V_O^{\cdot\cdot} - Ni_{Ti}'' - V_O^{\cdot\cdot})^x$	1048	20	116	42

S.9 Experimental Details

Synthesis and characterization details on our STN06 sample can be found in our previous publication[26] and are reviewed briefly in the main text. Raman spectra were collected in a backscattered illumination/observation geometry using a confocal WITec Alpha300 R Raman microscope (WITec, Germany) and a 532 nm (2.33 eV) 10 mW laser. All measurements were carried out with a 50X objective with a 9.1 mm working distance. *In situ* measurements were performed by heating an STN06 shard inside of a TS1500 Linkam stage (Resultec, Germany); this stage is equipped with a quartz window through which Raman measurements are taken. *In situ* heating was carried out under ambient atmosphere at temperatures ranging from 25 to 800 °C. The heating rate was 30 °C/min. After each target temperature was reached, the system was allowed to stabilize for one minute at that temperature, a Raman spectrum was collected, then the sample was heated to the next target temperature. The second quenching experiment was performed in a conventional furnace. The STN06 shard was placed into the furnace within an Al_2O_3 crucible. The sample was then heated under synthetic air at a ramp rate of 10 °C/min up to 900 °C, held at 900 °C for 10 hours, then quenched by removing the crucible from the furnace and quickly transferring the sample to a cold crucible. The small shard was allowed to briefly cool to room temperature before its Raman spectrum was collected.

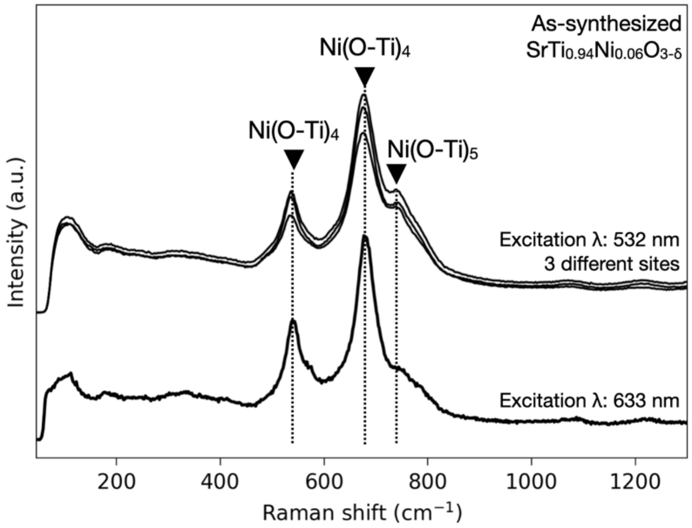

Figure S9. Raman spectra collected on the surface of the as-synthesized $Sr(Ti_{0.94}Ni_{0.06})O_{3-\delta}$ pellet at various sites and two different excitation wavelengths. The Raman shift of major bands was independent of excitation wavelength, indicating that these bands are in fact Raman bands and are not due to fluorescence.

S.10 Influence of Isolated Defects on Lattice Parameters

Table S4. Change in lattice parameters induced by various point defects calculated by DFT

Defect	a (Å)	Δa (Å)
None	3.940	
Ni_{Ti}''	3.955	+0.015
$(Ni_{Ti}'' - V_O^{\cdot\cdot})^x$	3.939	-0.001
$V_O^{\cdot\cdot}$	3.926	-0.014

To understand how individual defects influenced $SrTiO_3$'s lattice parameters, we carried out full structural minimizations on $3 \times 3 \times 3$ $SrTiO_3$ supercells. We treated a defect-free system as well as three defective systems, each of which contained a single instance of the point defects considered in this study. During structural minimizations, the cubic symmetry was fixed while all atomic positions and the lattice parameter a were free to relax. The optimized values for a are presented in **Table S4**.

Compared to the pure STO, the introduction of Ni^{2+} on the Ti sublattice expanded the lattice ($\Delta a = +0.015$). This is consistent with Ni^{2+}'s Shannon radius[38] in an octahedral environment (83 pm), which is considerably larger than Ti^{4+}'s (75 pm). Physically, Ni's lower oxidation state leads to weaker bonding between adjacent oxygens, increasing bond lengths and expanding the lattice. Meanwhile, introduction of a $V_O^{\cdot\cdot}$ into pure STO (plus two holes for charge compensation) resulted

in lattice contraction ($\Delta a = -0.014$). This, too, is intuitive, as the undercoordinated Ti^{4+} atoms will form stronger bonds with the remaining coordinated oxygens, contracting the surrounding lattice slightly. When Ni_{Ti}'' and a $V_O^{\cdot\cdot}$ were introduced together as $(Ni_{Ti}'' - V_O^{\cdot\cdot})^x$, however, the effects of the Ni_{Ti}'' and $V_O^{\cdot\cdot}$ on the lattice nearly cancel out, resulting in a very slight contraction of the lattice ($\Delta a = -0.001$). Previous XRD analyses[26] on our STN06 sample revealed a lattice parameter of $a = 3.902$ Å, only slightly contracted compared to pure STO's experimental lattice parameter ($a = 3.905$ Å). This is consistent with Ni_{Ti}'' and $V_O^{\cdot\cdot}$'s being present in roughly equal quantities.

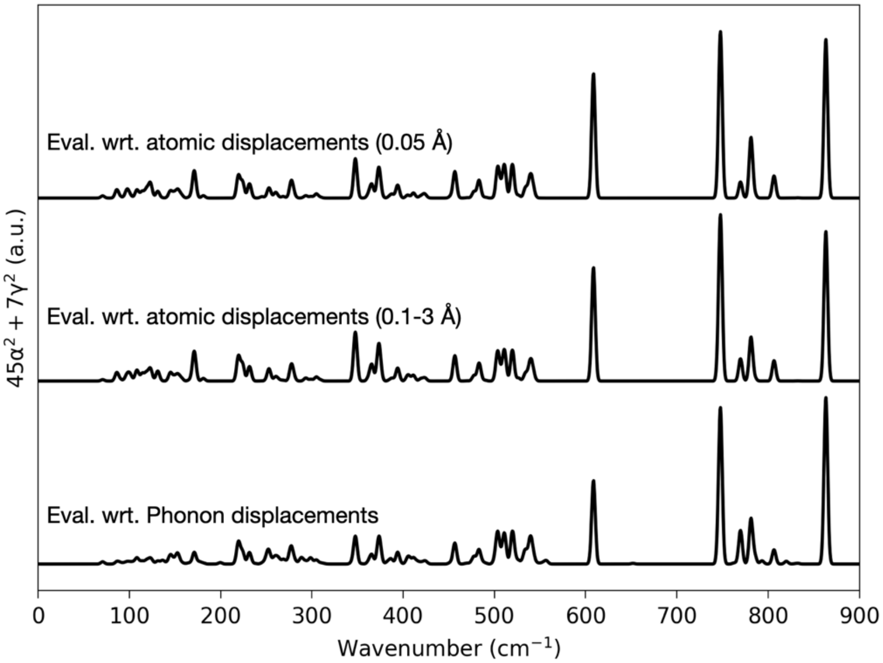

Figure S10. Total simulated Raman spectra of $3 \times 3 \times 3$ STO supercell containing a single $V_O^{\cdot\cdot}$, calculating with respect to atomic displacements as well as conventional phonon displacements. Results are in good agreement. We attribute slight intensity differences to numerical artifacts. To aid in visualization, discrete peaks were convoluted by Gaussians with standard deviation of 2 cm^{-1}.

References

(1) Bianchi, U.; Kleemann, W.; Bednorz, J. G. Raman Scattering of Ferroelectric $Sr_{1-x}Ca_xTiO_3$, x=0.007. *J. Phys. Condens. Matter* **1994**, *6* (6), 1229. https://doi.org/10.1088/0953-8984/6/6/025.
(2) Tkach, A.; Vilarinho, P. M.; Kholkin, A. L.; Pashkin, A.; Samoukhina, P.; Pokorny, J.; Veljko, S.; Petzelt, J. Lattice Dynamics and Dielectric Response of Mg-Doped $SrTiO_3$

Ceramics in a Wide Frequency Range. *J. Appl. Phys.* **2005**, *97* (4), 044104. https://doi.org/10.1063/1.1849822.

(3) Tenne, D. A.; Gonenli, I. E.; Soukiassian, A.; Schlom, D. G.; Nakhmanson, S. M.; Rabe, K. M.; Xi, X. X. Raman Study of Oxygen Reduced and Re-Oxidized Strontium Titanate. *Phys. Rev. B* **2007**, *76* (2), 024303. https://doi.org/10.1103/PhysRevB.76.024303.

(4) Vračar, M.; Kuzmin, A.; Merkle, R.; Purans, J.; Kotomin, E. A.; Maier, J.; Mathon, O. Jahn-Teller Distortion around Fe^{4+} in $SrFe_xTi_{1-x}O_{3-\delta}$ from X-Ray Absorption Spectroscopy, X-Ray Diffraction, and Vibrational Spectroscopy. *Phys. Rev. B* **2007**, *76* (17), 174107. https://doi.org/10.1103/PhysRevB.76.174107.

(5) Rabuffetti, F. A.; Kim, H.-S.; Enterkin, J. A.; Wang, Y.; Lanier, C. H.; Marks, L. D.; Poeppelmeier, K. R.; Stair, P. C. Synthesis-Dependent First-Order Raman Scattering in $SrTiO_3$ Nanocubes at Room Temperature. *Chem. Mater.* **2008**, *20* (17), 5628–5635. https://doi.org/10.1021/cm801192t.

(6) Tenne, D. A.; Farrar, A. K.; Brooks, C. M.; Heeg, T.; Schubert, J.; Jang, H. W.; Bark, C. W.; Folkman, C. M.; Eom, C. B.; Schlom, D. G. Ferroelectricity in Nonstoichiometric SrTiO3 Films Studied by Ultraviolet Raman Spectroscopy. *Appl. Phys. Lett.* **2010**, *97* (14), 142901. https://doi.org/10.1063/1.3499273.

(7) Phuong, D. T. T.; Hong, L. V.; Minh, N. V. $SrTi_{1-x}Ni_xO_3$ Nanoparticles: Synthesis and Characterisation. *Int. J. Nanotechnol.* **2011**, *8* (3/4/5), 312. https://doi.org/10.1504/IJNT.2011.038208.

(8) Yao, D.; Zhou, X.; Ge, S. Raman Scattering and Room Temperature Ferromagnetism in Co-Doped $SrTiO_3$ Particles. *Appl. Surf. Sci.* **2011**, *257* (22), 9233–9236. https://doi.org/10.1016/j.apsusc.2011.04.039.

(9) Chapron, D.; Cordero, F.; Fontana, M. D. Characterization of Oxygen Vacancies in $SrTiO_3$ by Means of Anelastic and Raman Spectroscopy. *J. Appl. Phys.* **2019**, *126* (15), 154101. https://doi.org/10.1063/1.5115106.

(10) Sediva, E.; Defferriere, T.; Perry, N. H.; Tuller, H. L.; Rupp, J. L. M. In Situ Method Correlating Raman Vibrational Characteristics to Chemical Expansion via Oxygen Nonstoichiometry of Perovskite Thin Films. *Adv. Mater.* **2019**, *31* (33), 1902493. https://doi.org/10.1002/adma.201902493.

(11) Ahmed, S.; Sarwar Hossain Faysal, A. K. M.; Khan, M. N. I.; Basith, M. A.; Shahriar Bashar, M.; Das, H. N.; Hasan, T.; Ahmed, I. Room Temperature Ferroic Orders in Zr and (Zr, Ni) Doped $SrTiO_3$. *Results Phys.* **2021**, *31*, 104940. https://doi.org/10.1016/j.rinp.2021.104940.

(12) Dwij, V.; De, B. K.; Tyagi, S.; Sharma, G.; Sathe, V. G. Raman Spectroscopic Investigation of Relaxor Behavior in Pr Doped $SrTiO_3$ and Origin of Fano Resonance. 11.

(13) Evarestov, R. A.; Blokhin, E.; Gryaznov, D.; Kotomin, E. A.; Maier, J. Phonon Calculations in Cubic and Tetragonal Phases of $SrTiO_3$: A Comparative LCAO and Plane-Wave Study. *Phys. Rev. B* **2011**, *83* (13), 134108. https://doi.org/10.1103/PhysRevB.83.134108.

(14) Rusevich, L. L.; Kotomin, E. A.; Zvejnieks, G.; Popov, A. I. *Ab Initio* Calculations of Structural, Electronic and Vibrational Properties of $BaTiO_3$ and $SrTiO_3$ Perovskite Crystals with Oxygen Vacancies. *Low Temp. Phys.* **2020**, *46* (12), 1185–1195. https://doi.org/10.1063/10.0002472.

(15) Krasnenko, V.; Rusevich, L. L.; Platonenko, A.; Mastrikov, Y. A.; Sokolov, M.; Kotomin, E. A. Water Splitting on Multifaceted $SrTiO_3$ Nanocrystals: Calculations of Raman Vibrational Spectrum. *Materials* **2022**, *15* (12), 4233. https://doi.org/10.3390/ma15124233.

(16) Perdew, J. P.; Burke, K.; Ernzerhof, M. Generalized Gradient Approximation Made Simple. *Phys. Rev. Lett.* **1996**, *77* (18), 3865–3868. https://doi.org/10.1103/physrevlett.77.3865.
(17) Kresse, G.; Furthmüller, J. Efficiency of Ab-Initio Total Energy Calculations for Metals and Semiconductors Using a Plane-Wave Basis Set. *Comput. Mater. Sci.* **1996**, *6* (1), 15–50. https://doi.org/10.1016/0927-0256(96)00008-0.
(18) Kresse, G.; Hafner, J. Ab Initiomolecular Dynamics for Liquid Metals. *Phys. Rev. B* **1993**, *47* (1), 558–561. https://doi.org/10.1103/physrevb.47.558.
(19) Kresse, G.; Hafner, J. Ab Initiomolecular-Dynamics Simulation of the Liquid-Metal–Amorphous-Semiconductor Transition in Germanium. *Phys. Rev. B* **1994**, *49* (20), 14251–14269. https://doi.org/10.1103/physrevb.49.14251.
(20) Kresse, G.; Joubert, D. From Ultrasoft Pseudopotentials to the Projector Augmented-Wave Method. *Phys. Rev. B* **1999**, *59* (3), 1758–1775. https://doi.org/10.1103/physrevb.59.1758.
(21) Blöchl, P. E. Projector Augmented-Wave Method. *Phys. Rev. B* **1994**, *50* (24), 17953–17979. https://doi.org/10.1103/physrevb.50.17953.
(22) Grimme, S.; Ehrlich, S.; Goerigk, L. Effect of the Damping Function in Dispersion Corrected Density Functional Theory. *J. Comput. Chem.* **2011**, *32* (7), 1456–1465. https://doi.org/10.1002/jcc.21759.
(23) Dudarev, S. L.; Botton, G. A.; Savrasov, S. Y.; Humphreys, C. J.; Sutton, A. P. Electron-Energy-Loss Spectra and the Structural Stability of Nickel Oxide: An LSDA+U Study. *Phys. Rev. B* **1998**, *57* (3), 1505–1509. https://doi.org/10.1103/physrevb.57.1505.
(24) Wang, L.; Maxisch, T.; Ceder, G. Oxidation Energies of Transition Metal Oxides within the GGA+U Framework. *Phys. Rev. B* **2006**, *73* (19), 195107. https://doi.org/10.1103/PhysRevB.73.195107.
(25) Hu, Z.; Metiu, H. Choice of U for DFT+U Calculations for Titanium Oxides. *J. Phys. Chem. C* **2011**, *115* (13), 5841–5845. https://doi.org/10.1021/jp111350u.
(26) O'Leary, W.; Giordano, L.; Park, J.; Nonnenmann, S. S.; Shao-Horn, Y.; Rupp, J. L. M. Influence of Sr-Site Deficiency, Ca/Ba/La Doping on the Exsolution of Ni from $SrTiO_3$. *J. Am. Chem. Soc.* **2023**, *145* (25), 13768–13779. https://doi.org/10.1021/jacs.2c12011.
(27) Monkhorst, H. J.; Pack, J. D. Special Points for Brillouin-Zone Integrations. *Phys. Rev. B* **1976**, *13* (12), 5188–5192. https://doi.org/10.1103/physrevb.13.5188.
(28) Baroni, S.; Resta, R. Ab Initio Calculation of the Macroscopic Dielectric Constant in Silicon. *Phys. Rev. B* **1986**, *33* (10), 7017–7021. https://doi.org/10.1103/PhysRevB.33.7017.
(29) Gajdoš, M.; Hummer, K.; Kresse, G.; Furthmüller, J.; Bechstedt, F. Linear Optical Properties in the Projector-Augmented Wave Methodology. *Phys. Rev. B* **2006**, *73* (4), 045112. https://doi.org/10.1103/PhysRevB.73.045112.
(30) Eriksson, F.; Fransson, E.; Erhart, P. The Hiphive Package for the Extraction of High-Order Force Constants by Machine Learning. *Adv. Theory Simul.* **2019**, *2* (5), 1800184. https://doi.org/10.1002/adts.201800184.
(31) Jinnouchi, R.; Karsai, F.; Verdi, C.; Asahi, R.; Kresse, G. Descriptors Representing Two- and Three-Body Atomic Distributions and Their Effects on the Accuracy of Machine-Learned Inter-Atomic Potentials. *J. Chem. Phys.* **2020**, *152* (23), 234102. https://doi.org/10.1063/5.0009491.
(32) Jinnouchi, R.; Karsai, F.; Kresse, G. On-the-Fly Machine Learning Force Field Generation: Application to Melting Points. *Phys. Rev. B* **2019**, *100* (1), 014105. https://doi.org/10.1103/PhysRevB.100.014105.

(33) Jinnouchi, R.; Lahnsteiner, J.; Karsai, F.; Kresse, G.; Bokdam, M. Phase Transitions of Hybrid Perovskites Simulated by Machine-Learning Force Fields Trained on the Fly with Bayesian Inference. *Phys. Rev. Lett.* **2019**, *122* (22), 225701. https://doi.org/10.1103/PhysRevLett.122.225701.
(34) Thomas, M.; Brehm, M.; Fligg, R.; Vöhringer, P.; Kirchner, B. Computing Vibrational Spectra from Ab Initio Molecular Dynamics. *Phys. Chem. Chem. Phys.* **2013**, *15* (18), 6608–6622. https://doi.org/10.1039/C3CP44302G.
(35) Long, D. A. *The Raman Effect: A Unified Treatment of the Theory of Raman Scattering by Molecules*; Wiley: Chichester ; New York, 2002.
(36) Stirling, W. G. Neutron Inelastic Scattering Study of the Lattice Dynamics of Strontium Titanate: Harmonic Models. *J. Phys. C Solid State Phys.* **1972**, *5* (19), 2711. https://doi.org/10.1088/0022-3719/5/19/005.
(37) Blokhin, E.; Gryaznov, D.; Kotomin, E.; Evarestov, R.; Maier, J. A Comparative Hybrid DFT Study of Phonons in Several SrTiO3 Phases. *Integr. Ferroelectr.* **2011**, *123* (1), 18–25. https://doi.org/10.1080/10584587.2011.570591.
(38) Shannon, R. D. Revised Effective Ionic Radii and Systematic Studies of Interatomic Distances in Halides and Chalcogenides. *Acta Crystallogr. A* **1976**, *32* (5), 751–767. https://doi.org/10.1107/S0567739476001551.
